# Molecular mechanisms of activation and regulation of ANO1-Encoded Ca^2+^-Activated Cl^-^ channels

**DOI:** 10.1080/19336950.2021.1975411

**Published:** 2021-09-27

**Authors:** M. B. Hawn, E. Akin, H.C. Hartzell, I. A. Greenwood, N. Leblanc

**Affiliations:** a Department of Pharmacology and Center of Biomedical Research Excellence for Molecular and Cellular Signal Transduction in the Cardiovascular System, University of Nevada, Reno School of Medicine, Reno, United States; b Department of Cell Biology, Emory University School of Medicine, USA; c Department of Vascular Pharmacology, St. George’s University of London, UK

**Keywords:** Calcium-activated chloride channel, TMEM16A, anoctamin-1, ANO1, CaMKII, PIP_2_, regulation, phosphorylation, calcium binding, structure

## Abstract

Ca^2+^-activated Cl^−^ channels (CaCCs) perform a multitude of functions including the control of cell excitability, regulation of cell volume and ionic homeostasis, exocrine and endocrine secretion, fertilization, amplification of olfactory sensory function, and control of smooth muscle cell contractility. CaCCs are the translated products of two members (ANO1 and ANO2, also known as TMEM16A and TMEM16B) of the Anoctamin family of genes comprising ten paralogs. This review focuses on recent progress in understanding the molecular mechanisms involved in the regulation of ANO1 by cytoplasmic Ca^2+^, post-translational modifications, and how the channel protein interacts with membrane lipids and protein partners. After first reviewing the basic properties of native CaCCs, we then present a brief historical perspective highlighting controversies about their molecular identity in native cells. This is followed by a summary of the fundamental biophysical and structural properties of ANO1. We specifically address whether the channel is directly activated by internal Ca^2+^ or indirectly through the intervention of the Ca^2+^-binding protein Calmodulin (CaM), and the structural domains responsible for Ca^2+^- and voltage-dependent gating. We then review the regulation of ANO1 by internal ATP, Calmodulin-dependent protein kinase II-(CaMKII)-mediated phosphorylation and phosphatase activity, membrane lipids such as the phospholipid phosphatidyl-(4,5)-bisphosphate (PIP_2_), free fatty acids and cholesterol, and the cytoskeleton. The article ends with a survey of physical and functional interactions of ANO1 with other membrane proteins such as CLCA1/2, inositol trisphosphate and ryanodine receptors in the endoplasmic reticulum, several members of the TRP channel family, and the ancillary Κ^+^ channel β subunits KCNE1/5.

## Introduction

**Figure 1.**
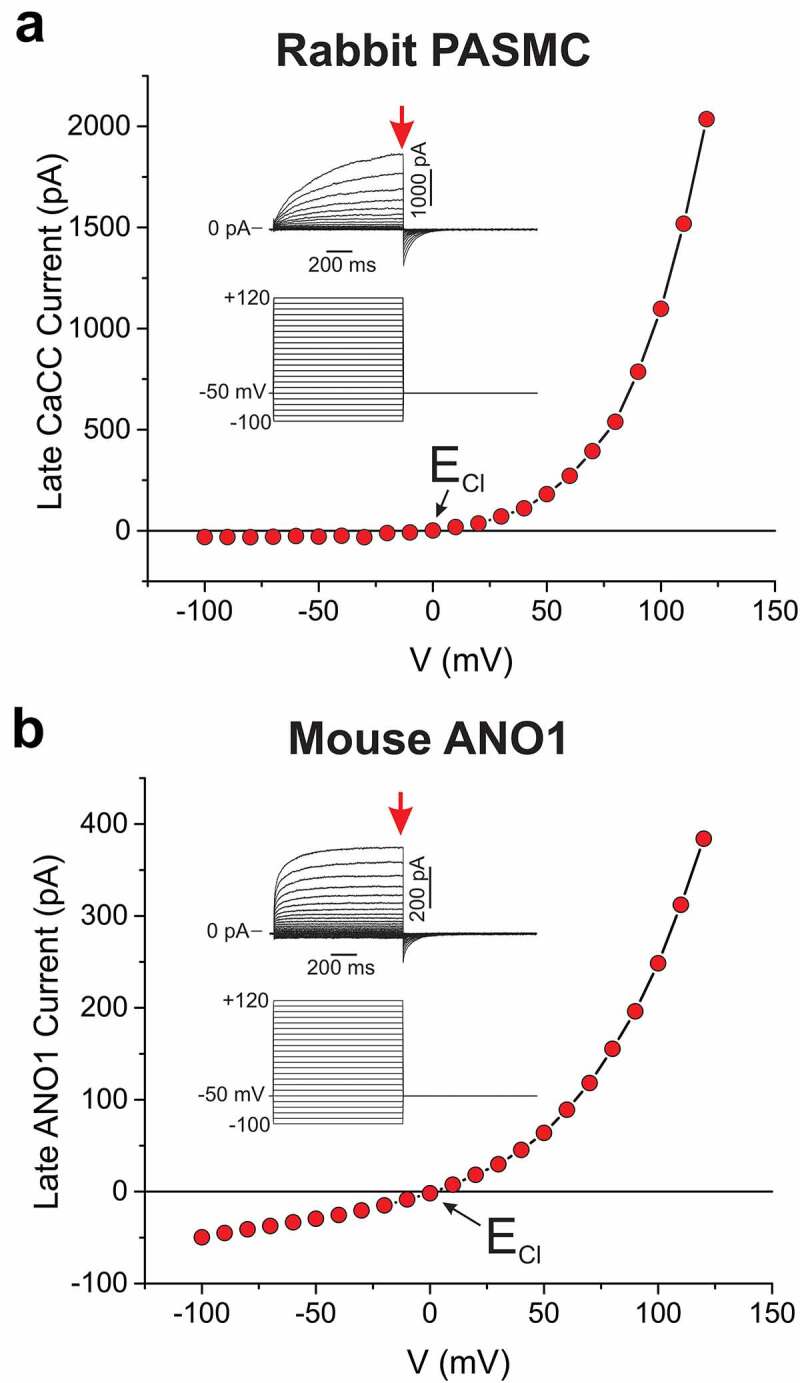
Typical experiments highlighting the similar biophysical properties of macroscopic Ca^2+^-activated Cl^−^ currents in native vascular smooth muscle cells and mouse ANO1 expressed in a mammalian cell line. (a) Current-voltage (i–v) relationship for late whole-cell Ca^2+^-activated Cl^−^ currents recorded from a freshly dissociated rabbit pulmonary artery smooth muscle cell (PASMC). *Inset*: the family of traces at the top were evoked by the voltage clamp protocol shown below from a holding potential of −50 mV. The red arrow indicates where the currents were measured to construct the I–V relationship. (b) I–V relationship for late Ca^2+^-activated Cl^−^ currents recorded in an HEK-293 cell transiently expressing mouse ANO1 (same clone as that used by Ayon *et al.* [177]). The nomenclature of this panel is identical to that in panel A. For both panels, the pipette solution was set to 500 nM free Ca^2+^ and contained 5 mM ATP. The exact composition of the bath and pipette solutions used in these experiments can be found in Wiwchar *et al.*[58] E_Cl_: predicted equilibrium potential for Cl

A chloride conductance activated by a physiological rise in intracellular Ca^2+^ concentration was first described in the early 1980s in *Xenopus* oocytes [1,2] and the retinas of salamanders [3]. Ιn oocytes, the underlying Ca^2+^-activated Cl^−^ channels (CaCC) triggered a membrane depolarization that inhibited polyspermy after fertilization through an undefined mechanism [4–6]. Ca^2+^-activated chloride currents (I_Cl(Ca)_) were subsequently recorded in many cell types including central and peripheral neurons [7–13], cardiac [14–16], skeletal [17] and smooth muscle [18–24] cells, epithelial cells [25,26], vascular endothelial cells [27,28], exocrine and endocrine gland cells [29–32], various types of leukocytes[33], mast cells[34], hepatocytes[35], and many others. CaCCs are anion-selective channels (anion permeability sequence of SCN^−^ > I^−^ > Br^−^ > Cl^−^ > gluconate) activated by an elevation in internal Ca^2+^ concentration ([Ca^2+^]_i_) above ~ 150 nM [36–42]. At physiological [Ca^2+^]_i_ (~ 250 nM to 1 µΜ), macroscopic I_Cl(Ca)_ display slow (hundreds of milliseconds to seconds) activation and deactivation kinetics and outward rectification as highlighted by the experiment in Figure 1a showing a typical family of whole-cell Ca^2+^-activated Cl^−^ currents recorded from a rabbit pulmonary artery smooth muscle cell dialyzed with 500 nM free Ca^2+^. Slow kinetics, outward rectification and a reversal potential near the equilibrium potential for Cl^−^ (E_Cl_) are hallmark properties of the native CaCCs of interest in this review.

Inside-out patch experiments in glomerular mesangial cells [43], endothelial cells [44], salivary acinar gland cells [45], cardiac cells [46], Xenopus oocytes [47], vascular smooth muscle cells [48–52], ventricular myocytes [46], and hepatocytes [35] indicated that CaCCs could be rapidly activated by an increase in Ca^2+^ concentration in the perfusate above ~ 150 nM. Single-channel experiments in these cell types revealed that CaCCs are small conductance channels (~ 3 pS) and, like macroscopic I_Cl(Ca)_ [11,53–59], they display outward rectification due to membrane depolarization promoting channel opening. This rectification is progressively alleviated by increasing the Ca^2+^ concentration on the inner side of the membrane ([Ca^2+^]_i_), so that the I–V relationship becomes almost linear at concentrations above 1 μM. The K_d_ for Ca^2+^-mediated channel opening decreases with membrane depolarization [32,44,53,59–61], an observation consistent with the notion that the binding site(s) probably lies within the transmembrane electric field. Two or three calcium ions were proposed to cooperatively regulate channel gating [32,59,60].

These currents are blocked by various structurally different anion transport inhibitors including CaCC inhibitors such as niflumic acid (NFA), anthracene-9-carboxylic acid (A9C), 5-nitro-2-(3-phenylpropylamino)benzoic acid (NPPB), and 4,4ʹ-diisothiocyano-2,2ʹ-stilbenedisulfonic acid (DIDS). The biophysical (Ca^2+^-, voltage- and time-dependence, low unitary conductance) and pharmacological properties of these currents define the so-called “classical” CaCCs, which are the ones of interest in this article. This is important because other genes, as will be discussed below, have also been proposed to encode classical CaCCs (e.g., Bestrophins, CLCA1, Tweety), but their profiles differ in several aspects from those mentioned above.

CaCCs play a crucial role in regulating the excitability of many types of smooth muscle cells and certain types of neurons, the control of fluid secretion by epithelial cells, olfactory transduction, and photoreceptor light responses. Most cell types maintain a Nernst potential for Cl^−^ that is more positive than the resting potential [62]. Consequently, activation of CaCCs in these cell types leads to Cl^−^ efflux and membrane depolarization. In vascular smooth muscle cells as an example, the membrane depolarization triggered by CaCCs causes activation of voltage-gated Ca^2+^ channels, Ca^2+^ influx and contraction [36,38–40,42]. An exception is mature neurons in which activation of CaCCs may produce stabilization or hyperpolarization of the resting membrane potential [63] (similar to GABA_A_ ligand-gated receptors) because E_Cl_ is near or negative to V_m_.

In 2008, two members of the Anoctamin gene family, Anoctamin-1 (ANO1) and 2 (ANO2), were identified as the molecular correlates of native CaCCs. The identification of ANO1 and ANO2 enabled investigations into their biophysical and pharmacological properties at the molecular level. This review focuses on recent advances in understanding mechanisms of activation and regulation of ANO1 channels and how these findings correlate with understanding the structures of Anoctamins. Importantly, we compare features from over-expression studies and observations made for CaCCs recorded in their native environment. Excellent reviews documenting the properties of native CaCCs [36–42,64–69] and Anoctamins are available [39,42,70–81]. This review specifically surveys recent findings in regard to the structure of ANO1, the molecular mechanisms involved in Ca^2+^-dependent activation of its gating, how alternative splicing influences its expression and function, and how different mediators regulate channel activity.

## Search for the molecular identity of native CaCCs

During the search for CaCC genes, members of no less than five structurally unrelated families of genes were proposed as molecular candidates for the “classical” Ca^2+^-activated Cl^−^ currents recorded in *Xenopus* oocytes [47,60], smooth [23,38,54–59,82–86] and skeletal muscle cells [17], parotid acinar [32] and lacrimal gland cells [30], and interstitial cells of Cajal in the gut [87]. These families of structurally unrelated proteins include: the CLCA family (ChLoride channels Calcium Activated; now known as “Chloride Channel Accessory”) [88–92], the long human isoform variant of CLC-3 (a voltage-gated Cl^−^ channel superfamily member that requires CaMKII for activation [37,93,94]), the products of human genes related to the Drosophila flightless locus called *Tweety* [95,96], Bestrophins (BEST1 and BEST2) [97,98], and more recently the TMEM16 or Anoctamin channel protein family [72,99–101].

Many investigators questioned the evidence supporting that CLCAs are *bona fide* transmembrane proteins capable of directly supporting ion transport. Analysis of hydropathy plots of the various CLCA protein members showed profiles that were unconventional for ion channel proteins: they lacked hydrophobic α-helices capable of forming transmembrane domains (for a review, see Loewen and Forsyth [102]). Moreover, CLCA proteins were found to exhibit similarity with surface adhesion proteins [89,103] and some members were secreted as truncated soluble proteins [104–106]. The idea of CLCAs forming transmembrane ion channels was later put to rest by experiments demonstrating that in Caco-2 lung epithelial cells that lacked an endogenous Ca^2+^-activated Cl^−^ conductance during differentiation, the expression of pCLCA1 failed to restore CaCC conductance [107]. More recent reports discussed in a section below now support the concept that CLCA may instead serve as accessory proteins because at least two members of this protein family were shown to upregulate ANO1-encoded CaCCs.

**Figure 2.**
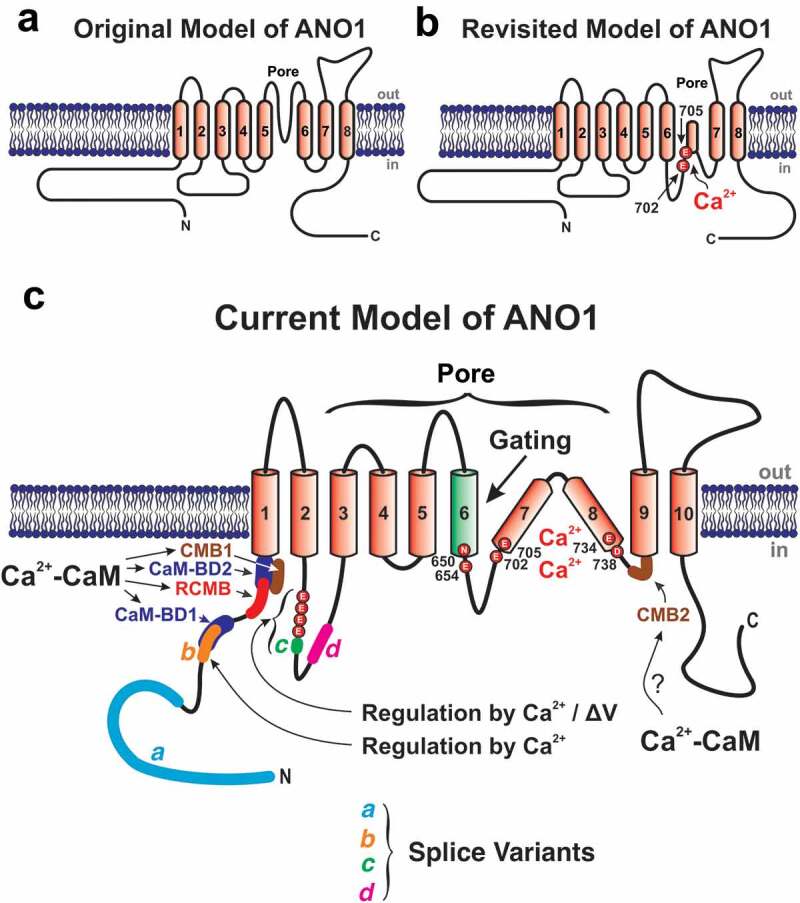
Proposed secondary structures of ANO1 and important domains determining its biophysical properties and interactions with Ca^2+^ and Calmodulin (CaM). (a) This is the originally proposed topology of ANO1, which was thought to comprise eight transmembrane domains, with the N- and C-terminal ends located intracellularly, and a pore region located between TMD5 and TMD6 and characterized by a reentrant loop [51,52][change references to 99, 100]. (b) Revised model of ANO1 based on mapping experiments by Yu *et al.*[124][change reference to 154] showing that certain amino acids originally thought to lie on the extracellular side of the membrane near TMD6 turned out to be located on the cytoplasmic side of the membrane. The model still comprised eight TMDs, but included a large cytoplasmic loop following TMD6 that reinserted part ways in the membrane to then reach TMD7. Two glutamate residues (E702 and E705) in close proximity from each other within the larger reinsertion loop were found to be critical for Ca^2+^ binding, a result that was later confirmed by another group [125][change reference to 155]. (c) Most recent consensus secondary structure of ANO1 that now comprises ten instead of eight TMDs. The diagram indicates the approximate position of the four alternatively spliced variants *a, b, c* and *d*, and the six amino acids (N650, E654, E702, E705, E734 and D738) postulated to coordinate the binding of two Ca^2+^ ions within each ANO1 monomer (see text for explanations). Please note that the positions of the labeled amino acids are relative to those of mouse ANO1-*ac*, which comprises 960 amino acids (NCBI sequence: NP_848747.5). The illustration also highlights the widespread localization of the pore between TMD3 and TMD8, the role of TMD6 in ANO1 activation following Ca^2+^ binding, the stretch of four consecutive glutamate residues immediately preceding splice variant *c* (EAVK) and hypothesized to modulate the Ca^2+^- and voltage-(ΔV)-dependence of ANO1, and splice variant *b* regulating the Ca^2+^-dependence of ANO1 (see text for explanations). Finally, the diagram shows the location of several color-coded calmodulin (CaM) binding sites in the N-terminal domain and short intracellular loop between TMD8 and TMD9. Some of these sites were proposed based on bioinformatics analysis while others were confirmed in biochemical assays. CaM-BD1 (proposed role: channel opening) and BD2 (proposed role: none?): Calmodulin Binding Domains 1 and 2 [129];[change reference to 161] RCMB: Regulatory Calmodulin-Binding Motif (proposed role: channel opening) [130];[change reference to 162] CMB1 and 2 (proposed role for both: increased permeability of ANO1 to HCO_3_^−^ relative to Cl^−^): Calmodulin Binding Motifs 1 and 2 [131][change reference to 163]

At the turn of the new millennium, a new hypothesis surfaced suggesting that the third member of the CLC family of Cl^−^ channels, CLC-3, is phosphorylated by CaMKII and is required for channel activity [93,94]. The possibility of CaMKII-activated CLC-3 as a molecular candidate for native classical CaCCs was also discarded because: 1) their biophysical properties (voltage- and time-independent) do not match those of native CaCCs (slow activation kinetics and deactivation kinetics, voltage-dependent; Figure 1a); 2) CLC-3 is now considered to be an 2Cl^−^/H^+^ exchanger primarily located in endolysosomal membranes instead of a plasma membrane ion channel [108]; 3) unlike CLC-3, native CaCCs are down-regulated by CaMKII-induced phosphorylation (see section below); and 4) native CaCCs are robustly activated by patch excision into a solution containing Ca^2+^, but no ATP nor CaMKII. Furthermore, a large body of evidence has suggested that native CaCCs are activated by a direct interaction of Ca^2+^ with binding sites on the cytoplasmic face of CaCCs.

The idea that the *Tweety* family of Cl^−^ channels encoded classical CaCCs was also rapidly dismissed because their high single channel conductance was more than 100 pS [95,96], compared to 3 pS for native CaCCs. Although the Bestrophins are a family of Ca^2+^-activated Cl^−^ channels (reviewed by Hartzell *et al.* [98]), their characteristics are different from classical CaCCs. They are similar to classical CaCCs with low single channel conductance (0.26–2.0 pS), a lyotropic permeability sequence of SCN^−^ > I^−^ > Br^−^ > Cl^−^ > F^−^, and a similar pharmacological profile. But, the *K_d_* for Ca^2+^ is ~ 200 nM and the currents of the four mammalian Bestrophin paralogs are time- and voltage-independent. Such properties contrast with I_Cl(Ca)_ recorded in *Xenopus* oocytes, smooth muscle cells, sensory neurons, and secretory epithelial cells, which display outward rectification at [Ca^2+^]_i_ < ~ 1 μM, and are time- and voltage-sensitive. Another argument against Bestrophins encoding classical CaCCs was the observation that in rat mesenteric arterial smooth muscle cells, the Ca^2+^- and cGMP-sensitive but voltage-insensitive Bestrophin 3 current coexists with the classical time- and voltage-dependent CaCC current [109–113].

## TMEM16/anoctamins as the long sought molecular candidates for CaCCs

The cloning of TMEM16 proteins, so-called Anoctamins, by three independent groups in 2008 [99-101] paved the way for numerous subsequent studies to determine the structural elements responsible for anion transport across the membrane and channel gating by intracellular Ca^2+^ and transmembrane voltage. ANO1 and ANO2 were established as the channels underlying Ca^2+^-activated Cl^−^ currents since they recapitulate the biophysical and pharmacological properties of native Ca^2+^-activated Cl^−^ currents (I_Cl(Ca)_) when expressed in mammalian cell lines [99–101,114,115]. An example of such currents produced by the expression of recombinant mouse ANO1 in HEK-293 cells is illustrated in Figure 1b. Similar to native CaCC currents recorded in vascular smooth muscle cells (Figure 1a), ANO1 currents recorded under identical conditions activate and deactivate slowly during step depolarizations and repolarizations, display outward rectification, and reverse near E_Cl_.

The first topology proposed for ANO1, based on hydropathy analysis, had eight transmembrane domains (TMD) with the C- and N-termini located intracellularly (Figure 2a) [42,70–73]. Investigators speculated that ANO1 contained a large intracellular loop between TMD1 and 2 and a reentrant loop, similar to the pore-loop of cation-permeable channels, between TMD5 and 6 that was suggested to form the anion-selective pore of the channel. The 8 transmembrane domain structure was later determined to be incorrect as structural studies later demonstrated the presence of 10 transmembrane domains as discussed in detail in a section below.

## Regulation of ANO1 Function by alternative splicing

Caputo *et al.* [99] described four alternative spliced variants labeled *a, b, c*, and *d*, depicted in Figure 2c. All four variants regulate the biophysical properties of ANO1, and its pharmacology [116]. Splice segment *a*, which starts the protein at the N-terminal end, is under the control of an alternative promoter. With few exceptions, this exon is constitutively expressed in nearly all tissues expressing ANO1. The role of this variant has not been fully elucidated but is likely involved in expression at the plasma membrane.

Splice segment *b* is located distally from splice segment *a* in the N terminus. It is encoded by exon 6b in human and mouse [117], and the translated product comprises 22 amino acids. Expression of this splice variant reduced the Ca^2+^ sensitivity of ANO1[114], but the mechanism responsible for this effect still remains unclear.

Splice variant segment *c*, encoded by exon 13 in human and mouse [117], comprises only four amino acids (EAVK). The short peptide segment is located in the first intracellular loop (Figure 2c). With the exception of brain and skeletal muscle, this splice variant is expressed in nearly all tissues [114]. Although it was initially speculated to play an exclusive role in altering the voltage- and time-dependence of ANO1 [114], another study by Xiao *et al.* [118] showed that its inclusion also influenced its Ca^2+^ sensitivity as reviewed in detail in the next section.

A short distance distal from splice variant *c* in the first intracellular loop lies splice variant *d*, which is a stretch of 26 amino acids encoded by exon 15 in human and mouse [117]. While earlier studies suggested that this peptide segment produced little to no effect on ANO1 function [99,114], a subsequent study showed that its inclusion decelerated both activation and deactivation kinetics [119].

Ferrera *et al.* [120] expressed a “minimal” isoform of ANO1 (called TMEM16(0)) that lacked all four splice variants. Expression of this isoform produced robust Ca^2+^-activated Cl^−^ currents. However, these currents were voltage- and time-independent, and displayed altered permeation and selectivity to both anions and cations. The strategy used by Ferrera *et al.* [120] to exclude splice segment *a* was to introduce a stop codon in lieu of the first ATG codon initiating translation. The same group later reported that this approach was erroneous because expression of an isoform truly lacking segment *a* produced no detectable channel activity [121]. They discovered that the discrepancy was caused by the existence of a non-canonical start codon (non-ATG) 5ʹ to the second ATG codon. This non-canonical start codon was presumed to be the start of the translated “minimal” ANO1 protein. Successive stepwise truncations of the N-terminal domain produced currents that were progressively smaller, highlighting the important role played by this domain, and segment *a* in particular, in protein trafficking and surface expression of ANO1. The non-traditional codon, possibly CTG, was shown to be physiologically relevant as a naturally occurring truncated protein consistent with this new start site was identified in human testis [121].

Next-generation RNA sequencing of human stomach revealed the existence of a novel exon that is upstream of exon 1 encoding for splice variant *a* and was therefore labeled exon 0 [122]. The inclusion of exon 0, which has 40 additional amino acids in human ANO1 (56 or 57 in mouse depending on NCBI sequences), enhanced ANO1 current. A novel promoter upstream of exon 0 was also identified and shown to be regulated by the cytokine interleukin-4 acting via the STAT6 transcription factor. This pathway is known to be involved in enhanced ANO1 expression in several forms of cancer [123].

Another level of complexity was revealed by Mazzone *et al.* [119] who found a novel variant in human stomach where ANO1 is predominantly expressed in interstitial cells of Cajal to regulate pacemaker activity and smooth muscle motility. This variant lacked exons 1 and 2 and part of exon 3. Its expression was increased in tissues from patients diagnosed with diabetic or idiopathic gastroparesis. When expressed in HEK-293 cells, this variant led to reduced and slower ANO1 currents, a phenotype proposed to compromise pacemaker activity in gastroparesis.

An example of further fine tuning of ANO1 function through alternative splicing was the observation of a differential pattern of expression within the same fully assembled dimeric protein. Ohshiro *et al.* [124] suggested that ANO1 in murine portal vein smooth muscle cells can form both homo- and heterodimers composed of two monomeric proteins being the translated products of identical or distinct alternatively spliced transcripts (*abc* or *acd* splice segments).

These studies indicated that alternative splicing plays a critical role in the regulation of ANO1 and provided insight on how this process shapes functional responses in different cell types. However, they also suggested that we probably only scratched the surface of this regulation modality. Additional splicing exons have indeed been identified in mouse, which are conserved in human, whose functions will require investigation [117].

## ANO1 structure

Anoctamins can be subdivided into two major subgroups, which bears relevance to our understanding of their structure: 1) true CaCCs, that include ANO1 and ANO2 [99–101], and 2) Ca^2+^-activated lipid scramblases, which include ANO3, ANO4, ANO6, ANO7, and ANO9 [125,126]. Some other Anoctamins (especially yeast IST2 and mammalian ANO8) have been shown to participate in membrane–membrane junctions and may be involved in lipid transport between membrane systems. Lipid scramblases facilitate the bidirectional transport of lipids between the inner and outer leaflets of the plasma membrane. The best characterized mammalian Anoctamin displaying scramblase activity, ANO6, was shown to exhibit a dual function as both a Ca^2+^-activated anion [127–132] or nonselective cation channel [131,133], and a Ca^2+^-activated lipid scramblase [125,131,134–137]. A representation of the 3D architecture of Anoctamins first came from the seminal X-ray crystallography study of Brunner *et al.* [138] who described the structure of an ortholog of mammalian Anoctamins from the fungus *Nectria haematococca* (nhTMEM16) identified to be a Ca^2+^-activated lipid scramblase that also mediates nonspecific ion transport [139]. This dual lipid scramblase/ion channel activity was also found in another ancestral Anoctamin protein purified from the fungus *Aspergillus fumigatus* (afTMEM16) [140]. Brunner *et al.* [138] showed that each monomer of nhTMEM16 is comprised of 10 instead of 8 membrane-spanning α-helices, a transmembrane hydrophilic cavity facing the lipid bilayer involved in catalyzing phospholipid translocation, and a Ca^2+^-binding site lying within this cavity. Mutations of residues involved in Ca^2+^ activation in this region impaired scramblase activity in nhTMEM16 and anion channel activity in ANO1. The observation that the expression of chimeric ANO1 protein comprising a domain between TMD4 and TMD5 of ANO6 conferred scramblase activity to ANO1, which does not normally exhibit this activity, strengthened the idea that the hydrophilic cavity of Anoctamins can support scramblase activity and ion conduction [135].

**Figure 3.**
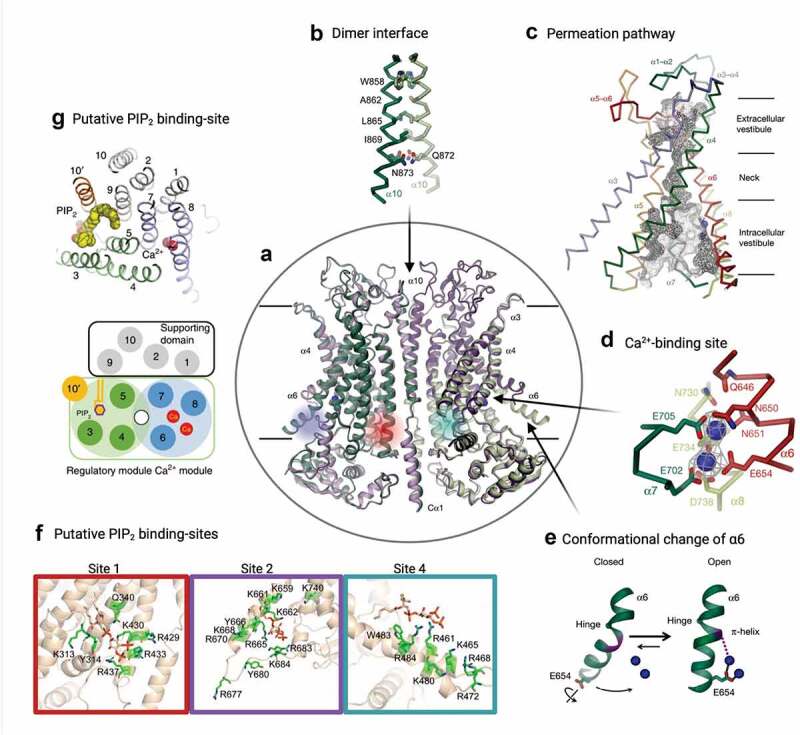
Key structural components of ANO1. (a-e) Panels modified from Paulino *et al*. (Reprinted by permission from Springer Nature Customer Service Center GmbH: Springer Nature, under license number 5110410425528) [138][change reference to 142]. (a) Ribbon representation of mTMEM16A(*ac*) viewed from within the membrane showing the Ca^2+^-bound (green) and Ca^2+^-free (violet) structures (PDB: 5OYB and 5OYG, respectively). The subunits are denoted with light and dark shades of the respective colors. The location of the Ca^2+^ in the Ca^2+^-bound structure are denoted by blue spheres. (b) Structure of the dimer interface composed of the extracellular part of α10. The side-chains of the interacting residues are shown as sticks. (c) A depiction of the ion conduction pore from one dimer of mTMEM16A, shown as a gray mesh. The extracellular vestibule narrows down to the neck region then opens back up into the intracellular vestibule. The location of Ca^2+^ binding is shown by the blue spheres. (d) Structure of the Ca^2+^-binding site (rotated 90° compared to a and c). The key amino acids involved in Ca^2+^ binding are denoted (amino acid numbers are shifted by 4 compared to the text due to the inclusion of the *c* splice variant). The mesh around the blue Ca^2+^ ions shows the cryo-EM density. (e) A depiction of the conformational changes of the inner half of α6 during channel activation due to Ca^2+^ binding. α6 is relaxed in the closed state. After binding of Ca^2+^, the helix rotates around the hinge to associate with α7 and α8 (as depicted in d) and is stabilized by interactions with the upper Ca^2+^ molecule. (f) Modified from Yu *et al.*[174] [change reference to 190]. Major sites shown to interact with PIP_2_. The amino acids composing each putative binding site are shown in green, with PIP_2_ shown in tan. The location on the ribbon structure is shown by a circle of the color around each site. Sites 1 and 2 are shown on subunit 1, while site 4 is shown on subunit 2. (g) Modified from Le *et al*. (Creative Commons license http://creativecommons.org/licenses/by/4.0/) [141][change reference to 150]. Top view of an ANO1 subunit with PIP_2_ depicted in yellow and bound Ca^2+^ shown as red spheres. α10 from the second monomer is shown as 10ʹ. The schematic depicts the two-module design proposed by Le *et al.*[141] whereby α3-5 form the PIP_2_-binding regulatory module (green), α6-8 form the Ca^2+^-binding module (blue), and α1, 2, 9, and 10 forming a supporting domain (gray). The permeation pathway is depicted by the white circle between the two modules. This putative PIP_2_ site is close to Site 4 identified in Yu *et al.*

A few years later, two groups [141–143] used Cryo-EM to decipher the structure of mouse ANO1. (For an in-depth review of Anoctamin structure-function, see Falzone *et al* [126] or Kalienkova *et al.* [144].) One group used a C-terminal truncation of the mouse ANO1 splice segment *a*[141], while the other group used the mouse ANO1 splice segment *ac* [142,143]. Both groups showed that Anoctamins are comprised of homodimers (Figure 3a and 3b), confirming earlier biochemical studies on ANO1 [145,146] and the structural study on nhTMEM16 [138]. Like nhTMEM16, each monomer contains 10 (Figure 2c and 3a) instead of the previously predicted 8 TMDs (Figure 2a and 2b). Each monomeric subunit bears an enclosed hydrophilic cavity surrounded by transmembrane helices TMD3-TMD8 that is presumed to be the anion permeation pathway (Figure 3c). Adjacent to the pores near the cytoplasmic side of the membrane are Ca^2+^-binding pockets that accommodate two Ca^2+^ ions and regulate the opening of the pore (Figure 3a and 3d). Each monomer is gated independently [147,148]. The permeation pathway is an hourglass shape where an outer vestibule narrows down to a smaller neck near the Ca^2+^ binding domain, then opens up again into a wider region on the intracellular side (Figure 3c). For all of the current cryo-EM structures including the structures with two Ca^2+^ ions bound that would be expected to show the channel in an activated state [141–143], the size of the narrowest part of the pore is too small to permit the flow of anions. These non-conducting structures would be expected because ANO1 undergoes time-dependent rundown during Ca^2+^ activation, which pushes the channel into an inactive state. Lam *et al.* [149] attempted to find conditions to maintain the active state of the channel during the cryo-EM process by purifying the protein in the presence of PIP_2_, shown to prevent or reverse Ca^2+^-induced channel insensitivity [150,151], combined with the addition of Ca^2+^ briefly before sample vitrification. They also examined the structure of the constitutively active mutant I551A in the presence of Ca^2+^[149]. Neither of these strategies produced a structure with a fully open pore, perhaps due to the detergent environment [152]. Molecular dynamics (MD) simulations of ANO1 showed a dilation of the pore upon binding of PIP_2_ that is consistent with an activated channel, supporting the idea that the proper lipid environment is essential to resolve a cryo-EM channel with a dilated pore [153]. However, these simulations were performed on a structure of ANO1 missing large portions of the N- and C-terminus. The N-terminus is known to be important in channel function.

## Hunt for the Ca^2+^-binding site(s)

Kinetic analysis of whole-cell Ca^2+^-activated Cl^−^ currents in native cells had previously revealed that the Ca^2+^ sensitivity of CaCCs is voltage-sensitive, with membrane depolarization reducing the apparent *K_d_* for Ca^2+^ in *Xenopus* oocytes [60], pancreatic acinar [32], and vascular smooth muscle [59] cells. Results obtained with expressed ANO1 were in agreement with such a property [154,155]. The voltage-dependence of the Ca^2+^ sensitivity arises from membrane potential influencing Ca^2+^ binding, although the possibility of a voltage-dependent transition step following Ca^2+^ binding could not be ruled out [144,149]. Biophysical analysis of the Ca^2+^-dependence of CaCCs suggested that two [32] or three [59,60] Ca^2+^ ions trigger channel activation. Expression studies using ANO1 recombinants also yielded Hill coefficients > 1, pointing to channel opening requiring more than one Ca^2+^ ion.

The discovery of anoctamins rapidly sets in motion an intense search of the Ca^2+^-binding site(s) responsible for their activation and how this process is influenced by membrane potential and permeating ions. Since the initial structure did not reveal obvious calcium-binding structures such as EF hands, investigators searched for negatively charged amino acid clusters that could serve as Ca^2+^-binding domains, similar to the “Ca^2+^ bowl” structure of BK channels. One such region in the 1^st^ intracellular loop is a stretch of four consecutive glutamate residues (Figure 2c), with a 5^th^ consecutive glutamate belonging to the very short alternatively spliced variant *c* (EAVK). Ferrera *et al.* [114] first showed that inclusion of segment *b*, a domain comprising 22 amino acids located in the cytoplasmic N-terminal domain of ANO1, reduced the Ca^2+^-sensitivity of ANO1 by ~ 4-fold. The same study also showed that deletion of segment *c*, which is expressed in most tissues [114], potently attenuated the characteristic time-dependent relaxation of ANO1 currents. These authors concluded that the *c* segment is involved in the voltage-dependence of ANO1. Xiao *et al.* [118] showed that removing the four consecutive glutamate residues abolished the voltage-dependence of ANO1 while having no effect on Ca^2+^ sensitivity. In contrast, omitting segment *c* considerably reduced the apparent affinity for Ca^2+^, but the currents were still voltage-dependent, at odds with those of Ferrera *et al.* [114]. Xiao *et al.* [118] suggested possible species differences or an underestimation of the role of Ca^2+^ in the conditions by Ferrera *et al.* [114] as ANO1 currents appeared to be comprised of both Ca^2+^-dependent and Ca^2+^-independent components. Despite evidence supporting a clear role by the segment EEEE(EAVK) in transducing Ca^2+^- and voltage-dependent sensing of ANO1, Xiao *et al.* [118] were skeptical about these residues being “the” Ca^2+^-binding site(s) responsible for gating because neutralizing the charge of the four glutamates did not affect the apparent Ca^2+^ sensitivity.

Yu *et al.* [154] proposed that E702 and E705 of mouse ANO1 expressing the *a* and *c* segments are two critical residues in determining Ca^2+^ sensitivity (Figure 2b and 2c). Mutating these two residues to glutamines profoundly reduced the apparent Ca^2+^ affinity while producing only modest effects on the voltage-dependence. The identification of these two glutamate residues serving as a potential Ca^2+^-binding site(s) required a significant modification of the membrane topology of ANO1. HA epitope tag mapping and cysteine scanning experiments confirmed that these two glutamates, previously thought to be located extracellularly, were instead facing the cytoplasmic side of the membrane (Figure 2b), which brought about uncertainty about the existence of the previously proposed reentrant loop.

Tien *et al.* [155] confirmed that E698 and E701 of mouse ANO1-*a*, corresponding to E702 and E705 in the mouse ANO1-*ac* clone (NCBI sequence: NP_848757.5) used by Yu *et al.* [154] (as labeled in Figure 2b and 2c), play a key role in mediating Ca^2+^ activation of ANO1. Moreover, they refined the revised model of Yu *et al.* [154] by proposing that E654, E734 and D738 (positions relative to mouse ANO1-*ac*; in Tien *et al.* [155] these three residues correspond to E650, E730 and D734), which are located in the vicinity of E702 and E705 (sequence includes variant *ac* or EAVK), form a spatially clustered metal ion-binding pocket responsible for the coordinated binding of Ca^2+^.

## Evidence for direct Ca^2+^ binding vs. activation by Ca^2+^-calmodulin

Direct binding of Ca^2+^ to promote channel opening was challenged by several groups that proposed instead that activation by Ca^2+^ is indirect via calmodulin. This proposal was reminiscent of studies on small conductance Ca^2+^-activated K^+^ channels (SK) whose activation is triggered by Ca^2+^ binding to CaM tethered to the channel [156–158]. As suggested by some investigators for native CaCCs prior to the discovery of Anoctamins [159,160], several groups proposed a similar paradigm for ANO1 even though this channel lacked the classical “IQ” signature binding sequence for CaM. Overall, studies suggesting that calmodulin is required for ANO1 activation are inconsistent and contradictory as detailed below.

Using bioinformatics, Tian *et al.* [161] postulated the existence of two novel CaM-binding domains (CaM-BD1 and CaM-BD2) on the N-terminal end of ANO1 (Figure 2c). They showed that while CaM-BD2 did not bind CaM and produced no effect on channel gating, CaM-BD1 was indispensable for transducing Ca^2+^ activation in the presence of internal Ca^2+^. One caveat to this assertion is that the CaM-BD1 sequence overlaps significantly with splice segment *b* (Figure 2c) and expression of ANO1 lacking this alternatively spliced sequence produces robust I_Cl(Ca)_ that are Ca^2+^- and voltage-dependent. Moreover, inclusion of segment *b*, which would be predicted to enhance CaM binding, reduces Ca^2+^ sensitivity[114]. Vocke *et al.* [162] documented the existence of a distinct CaM-binding domain on the N-terminal end of both ANO1 and ANO2, referred to as the “Regulatory Calmodulin-Binding Motif” or RCBM (Figure 2c). This domain was shown to be essential for activation of ANO1 and ANO2 by submicromolar Ca^2+^ concentrations, as well as for a slower inactivation of I_Cl(Ca)_ when the internal face of the membrane is exposed to cytoplasmic Ca^2+^ concentrations in the tens of micromolar or higher. A third report presented evidence for the existence of two additional CaM-binding domains called “Calmodulin-Binding Motifs” 1 and 2 (CBM1 and CBM2; Figure 2c) [163]. A large portion of CBM1 overlaps with CaM-BD2 on the N-terminus of ANO1 which Tian *et al.* [75] showed had no impact on channel activity. CBM2 was shown to be located in the short intracellular loop preceding TMD9. In contrast to the other two studies, Jung *et al.* [163] proposed that CaM is not involved in ANO1 activation by Ca^2+^ but instead alters the ion conduction pathway by increasing the permeability of the channel to HCO_3_^−^ relative to Cl^−^, a property ascribed to be important in regulating fluid secretion in submandibular acinar gland cells. In sharp contrast, another group examined this question in experiments carried out under well-controlled conditions and found no evidence of a shift in anion permeability in response to Ca^2+^ elevations[164].

The arguments against a role for Ca^2+^-CaM in channel activation are strong [155,165,166]. Ba^2+^, which does not bind CaM, can substitute for Ca^2+^ to activate ANO1 [166]. Co-immunoprecipitation experiments only revealed a weak association between CaM and ANO1[166]. Terashima *et al.* [165] convincingly demonstrated that purified ANO1 reconstituted in liposomes recapitulated the Ca^2+^-dependence of human ANO1-*abc* expressed in mammalian cell lines or endogenously in native cells. Moreover, the same two mutations (E724Q/E727Q) that led to a profound reduction in Ca^2+^ sensitivity (E702 and E705 in mouse ANO1-*ac*; Figure 2c) [154,155] also abrogated Ca^2+^ activation of purified ANO1. Together, these data support the idea that ANO1 activation occurs exclusively by direct Ca^2+^ binding to the Ca^2+^-binding site(s) identified in structural studies.

## Is ANO1 modulated by bound CaM?

Yang and Colecraft [167] concluded that the direct activation of ANO1 by Ca^2+^ is supported by an overwhelming body of evidence, which is further corroborated by structural studies (discussed below). Similar to SK channels, they found CaM to be tethered on the N-terminus of ANO1, even in the absence of intracellular Ca^2+^ (apoCaM). They proposed, based on their original study [168], that CaM is not the Ca^2+^ sensor but instead acts as a regulator of ANO1 channels, enhancing Ca^2+^ sensitivity at [Ca^2+^]_i_ < 1 μM (termed “Ca^2+^-dependent sensitization of activation” or CDSA) and decreasing channel activity at [Ca^2+^]_i_ > 10 μM through CaM-dependent inactivation or CDI. Deletion of splice segment *a* resulted in loss of binding of apoCaM and disappearance of both CDSA and CDI, while the exclusion of segment *b* selectively suppressed CDI. In conclusion, tethered or freely diffusing CaM is not required to activate the channels, but it is possible that CaM may act as a modulator of ANO1.

## Mechanisms involved in Ca^2+^-dependent activation of ANO1

Patch-clamp studies suggested that the voltage-dependence of the apparent *K_d_* for Ca^2+^ and Hill coefficient (> 2) of CaCCs in Xenopus oocytes [60], parotid acinar [32] and vascular smooth muscle [59] cells, as well as recombinant ANO1 expressed in HEK-293 cells [101], may arise, at least in part, from Ca^2+^ accessing a binding site within the transmembrane electric field. The cryo-EM structures confirmed that calcium ions must partially penetrate the transmembrane electric fields, approximately one-third of the thickness of the membrane on the intracellular side, to reach the Ca^2+^-binding site (Figure 3a). They also supported the idea of activation of ANO1 by direct binding of Ca^2+^ to the previously discovered Ca^2+^-binding pocket located within TMD6-TMD8 (N650, E654, E702, E705, E734, and D738 relative to the mouse ANO1-*ac* sequence; Figure 2c) [154,155,169]. Importantly, structures were solved for ANO1 with different levels of Ca^2+^ binding, including without Ca^2+^ bound or with either one or two Ca^2+^ ions bound. TMD3, TMD4, and TMD6 surrounding the neck region for Ca^2+^ free vs. Ca^2+^ bound structures displayed minor differences. However, the most pronounced differences in the alignment between the different levels of Ca^2+^ binding were in the intracellular half of TMD6 [141,142]. Since TMD6 contributes to both the pore and Ca^2+^- binding sites, it is poised to be a fundamental structure in the activation of Anoctamins. Indeed, it appears to undergo conformational changes upon the binding of Ca^2+^ playing a pivotal role in gating and ion permeation (Figure 3a and 3e). Peters *et al.* [169] identified G640 as a hinge by which TMD6 undergoes conformational changes during channel gating. Without Ca^2+^ bound, an aqueous pathway is accessible to the Ca^2+^ binding sites as TMD6 interacts with TMD7. Upon binding of Ca^2+^, TMD6 moves toward TMD4. The movement of TMD6 alters the size of the pore neck, likely contributing to the gating of the channel [142]. The rearrangement of TMD6 involves the formation of a π-helix from the α-helix conformation [142]. Two mutations, I637A and Q645A, enhance Ca^2+^ sensitivity, likely mimicking conformational changes associated with the binding of Ca^2+^[169]. In addition to the conformational change observed upon Ca^2+^ binding, the positive charge density of Ca^2+^ ions binding adjacent to the ion permeation path neutralizes negative charges, which enhances anion permeation [170].

Recent structures for the scramblases ANO6 (TMEM16F) and ANO10 (TMEM16K) revealed that these proteins contain a third Ca^2+^ binding site in TM2 and TMD10 [171,172]. Le and Yang [173] used electrophysiological, mutagenesis, and metal bridging experiments to demonstrate that the third Ca^2+^ binding site in ANO1 enhances Ca^2+^-dependent activation through a long-range allosteric mechanism. Based on the previously resolved ANO1 structures, analysis of electron density maps confirmed that the third-binding site could be present in ANO1 as well.

## Regulation of native CaCCs by CaMKII and serine-threonine phosphatases

Many reports published prior to the discovery of ANO1 suggested that kinases and phosphatases regulate native CaCCs. These ideas stemmed from several reports showing that the activity of CaCCs was unstable following patch excision. Experiments in airway epithelial [174] and vascular smooth muscle [50,51] cells revealed a very rapid rundown when transitioning from the cell-attached to the inside-out path clamp configuration with single channel activity usually disappearing within seconds to a few minutes. This property made it difficult for investigators to study the biophysical properties of the channels such as their Ca^2+^- and voltage-dependence. This was also observed for ANO1 expressed in mammalian cell lines [175].

The first convincing evidence for regulation of native CaCCs by post-translational modification came from a study in equine tracheal smooth muscle cells by Wang and Kotlikoff [84]. These investigators showed that Ca^2+^-activated Cl^−^ currents evoked by either a rapid exposure to caffeine, the purinergic agonist ATP, or the Ca^2+^ ionophore ionomycin, displayed a shorter time course than that of the Ca^2+^ transient measured simultaneously with the fluorescent Ca^2+^ indicator Fura-2. When exposed to the CaM inhibitor W7 or a specific inhibitor of CaMKII (KN-93 or peptide inhibitor), I_Cl(Ca)_ and Ca^2+^ transients followed a similar time course. Similar effects were observed when replacing intracellular ATP with the non-hydrolyzable form of ATP, AMP-PNP, which indicated that phosphorylation was probably playing a role in this process. Consistent with this hypothesis, inhibition of the Ca^2+^-independent phosphatases PP1 and PP2A by okadaic acid accentuated and accelerated I_Cl(Ca)_ inhibition while having only a minor effect on the Ca^2+^ transient. The authors postulated that elevation of intracellular Ca^2+^ levels might cause CaMKII-mediated phosphorylation resulting in channel closure or “inactivation”. They also proposed that such regulation would constitute an efficient negative feedback system to terminate post-synaptic transmission by opposing the sustained depolarization caused by CaCC activation.

Greenwood *et al.* [54] examined this question in rabbit arterial and venous smooth muscle cells using the whole-cell patch clamp configuration by clamping free intracellular Ca^2+^ concentration ([Ca^2+^]_i_). They showed that blocking CaMKII with KN-93 or Autocamtide-2-related inhibitory peptide (ARIP) increased the magnitude of I_Cl(Ca)_ in coronary and pulmonary artery myocytes dialyzed with [Ca^2+^]_i_ in the 500–1000 nM Ca^2+^ range. This effect was consistent with a modulation of the gating properties of the channels as evidenced by the noted acceleration of the slow current relaxation during depolarizing steps, slower deactivation during repolarizing steps, and a leftward shift in the steady-state activation curve. Cell dialysis with a constitutively active Ca^2+^-independent CaMKII isoform (AutoCaMKII) produced opposite effects.

Interestingly, in portal vein myocytes, such a mode of regulation by CaMKII was not detected, and CaMKII appeared to instead enhance I_Cl(Ca)_ in a small fraction of cells. Thus, the authors could only speculate that the differential effects of CaMKII-mediated phosphorylation of CaCCs in arterial and venous myocytes might be attributed to a differential pattern of CaMKII isoforms expression (and likely phosphatases) and/or CaMKII phosphorylating unidentified regulators of CaCCs in the two cell types.

**Figure 4.**
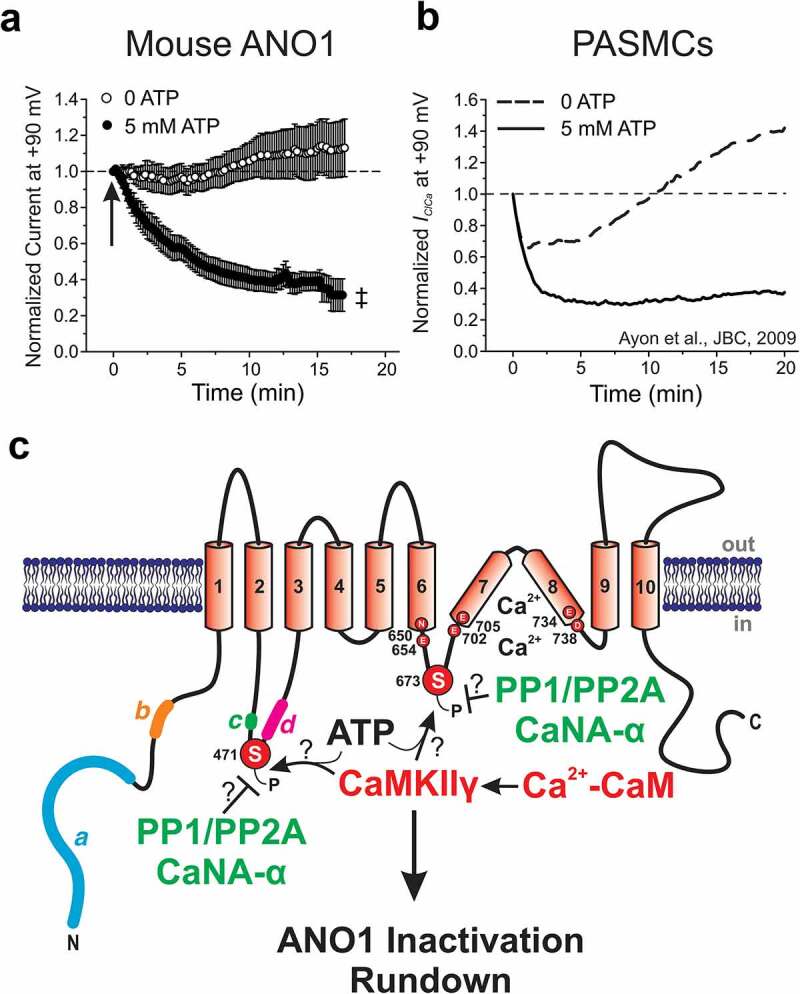
Regulation of ANO1 channels by ATP and calmodulin dependent protein kinase II (CaMKII)-mediated phosphorylation. (a) and (b) These two plots are reproduced from Ayon *et al.* [177] (panels A and B of their Figure 3 with a minor change to the title in panel A from “TMEM16A” to “Mouse ANO1” to reflect the main abbreviation used in this review for internal consistency) with permission from the *American Physiological Society©*. The two panels show the time course of changes in mean ± s.e.m. late Ca^2+^-activated Cl^−^ current amplitude recorded at +90 mV every 10 s from a holding potential of −50 mV. All currents were normalized to the initial current measured at time = 0, which corresponds to seal rupture (indicated by arrow in panel A) in the whole-cell configuration with a pipette solution set to 500 nM free Ca^2+^ to activate the channels, and 5 mM ATP (filled circles in panel A, n = 26; and continuous line in panel B) or 0 mM ATP (open circles in panel A, n = 14; and dashed line in panel B) to alter the state of global phosphorylation. Mouse ANO1: expression of mouse ANO1 (*a* variant) in HEK-393 cells; PASMCs: rabbit pulmonary artery smooth muscle cells. The plot in panel B was originally reproduced from *Ayon et al.* [57] with permission from the American Society for Biochemistry and Molecular Biology*©*. Panels A and B highlight the remarkable similarity in the response of ANO1 and native I_Cl(Ca)_ to intracellular ATP. ^‡^ Significant difference between the two groups (unpaired *t* test) with *P*< 0.001. (c) This diagram is reproduced from Figure 2c with minor modifications and again indicates the position of the four alternatively spliced variants *a, b, c* and *d*, and the six amino acids (N650, E654, E702, E705, E734 and D738, all related to mouse ANO1-*ac*; NCBI sequence: NP_848747.5) postulated to coordinate the binding of two Ca^2+^ ions within each ANO1 monomer (see text for explanations). It depicts the location of the two speculated sites (Serine 471 and Serine 673) for phosphorylation (denoted by the letter “P”) by the gamma isoform form of CaMKII (CaMKIIγ), which has been suggested to be responsible, at least in part, for ANO1 inactivation and rundown following seal rupture in the whole-cell patch clamp configuration in cells dialyzed with adenosine triphosphate (ATP). The figure also highlights the proximity of these two sites with splice variants *c* and *d*, and the postulated Ca^2+^ binding site, respectively. Finally, the diagram illustrates the possibility that type 1 and 2A protein phosphatases (PP1/PP2A) and/or the α isoform of calcineurin (CaNA-α; also referred to as protein phosphatase 2B) might be involved in dephosphorylating the two serine residues phosphorylated by CaMKIIγ

In a subsequent study, Ledoux *et al.* [55] reported that the Ca^2+^- and CaM-dependent serine-threonine phosphatase Calcineurin (CaN; PP-2B) exerted an opposite effect to that of CaMKII on I_Cl(Ca)_ in rabbit coronary artery myocytes by promoting channel opening. They found that blocking CaN with Cyclosporin A (CsA) or a specific CaN peptide inhibitor, which would result in a higher state of phosphorylation by CaMKII, reduced I_Cl(Ca)_ (Figure 4c). Inhibition of dephosphorylation by CsA reduced the Ca^2+^ sensitivity and activation kinetics of I_Cl(Ca)_, and increased deactivation kinetics. A subsequent study showed that cell dialysis with exogenous CaN-Aα (Figure 4c), but not CaN-Aβ, increased I_Cl(Ca)_ and altered its kinetics in rabbit pulmonary myocytes (PA) [56]. A report by Ayon *et al.* [57] further demonstrated that the modulation of I_Cl(Ca)_ by CaN in PA myocytes appeared to be upstream of another dephosphorylation step involving at least one Ca^2+^-independent phosphatase because the inhibition of PP1/PP2A led to similar effects to those produced by specific inhibitors of CaN (Figure 4c). The hypothesis of PP1 operating downstream of CaN was supported by the observation that the effect of an intracellular application of CaN-Aα was obliterated by the highly specific PP1 inhibitor NIPP-1 (0.1 nM).

To better understand the impact of phosphorylation on the biophysical properties of CaCCs in rabbit PA myocytes, Angermann *et al.* [59] examined the effects of dialyzing the cells with a pipette solution containing 3 mM ATP to support phosphorylation, or 0 mM ATP or 3 mM AMP-PNP, a non-hydrolyzable analog of ATP, to induce a global state of dephosphorylation. In the presence of ATP, I_Cl(Ca)_ ran down by ~80% over 20 min. In contrast, the current only ran down by ~45% during the first 2 min of cell dialysis in cells dialyzed with 3 mM AMP-PNP, but then slowly recovered to reach a level after 20 min that was similar to the initial current recorded after seal rupture. Omitting ATP from the pipette solution produced identical effects to AMP-PNP, suggesting that ATP binding *per se* did not appear to be involved in the inhibition of CaCCs in the presence of ATP. In addition, phosphorylation produced a marked decrease in voltage sensitivity while having little to no effect on the Ca^2+^-dependence. Marked effects on I_Cl(Ca)_ kinetics were also noted with dephosphorylation accelerating activation during strong depolarizing steps and slowing deactivation during repolarization, indicating a shift toward the open state. These studies demonstrated that native CaCCs are down-regulated by CaMKII-mediated phosphorylation, a process opposed by both Ca^2+^-dependent and Ca^2+^-independent phosphatases. Thus, in vascular smooth muscle cells, CaCC down-regulation would be expected to attenuate and/or abbreviate the Ca^2+^ transient triggered by vasoconstrictors coupled to G_q_-protein coupled receptors by promoting calcium channel closure near the resting membrane potential, most likely through a reduction in voltage sensitivity.

## Regulation of ANO1 by CaMKII-mediated phosphorylation

The first evidence for a possible regulation by phosphorylation of expressed ANO1 came from a study by Tian *et al.* [161] . In addition to proposing that intracellular CaM, ATP, and the actin cytoskeleton exerted a permissive role on ANO1 function, this group also showed that KN-62, a CaMKII blocker, augmented ANO1 currents evoked by ionomycin. Surprisingly, no explanation was offered about the more potent CaMKII inhibitor KN-93 being ineffective. Despite the identification of numerous putative phosphorylation sites for several kinases such as Protein Kinases A and C, Casein Kinase 2 and MAPK/ERK, and the serine/threonine phosphatases PP1/PP2A, ANO1 was found to be insensitive to broad spectrum and specific inhibitors of these enzymes. A subsequent study by Tian *et al.* [75] revealed that expressed ANO1 currents in HEK-293 cells ran down after seal rupture in the presence of internal ATP, and KN-62 antagonized this effect. In contrast, cell dialysis with exogenous constitutively active CaMKII inhibited ANO1 currents. Together, these preliminary studies suggested that, like vascular myocytes [54–57,59,84], ANO1 expressed in a heterologous expression cell system also appeared to be down-regulated or “inactivated” by a phosphorylation step involving CaMKII.

The next question concerned whether one or more CaMKII-mediated phosphorylation events directly target the pore-forming subunit of ANO1. Lin *et al.* [176] performed siRNA experiments in cultured mouse basilar artery smooth muscle cells (BASMC) and identified CaMKIIγ as the isoform responsible for down-regulating I_Cl(Ca)_ and ANO1 activity. They also proposed that S727 is the site phosphorylated by CaMKIIγ. The specific mouse splice variant isoform was not indicated, but the short sequence shown in their article was consistent with S673 in Figure 4c, which is relative to ANO1-*ac*. This site was interesting and relevant based on its proximity to the Ca^2+^ binding sites previously identified by other groups [154,155]. There were a few puzzling findings in this report. Expression of a mutant of ANO1 neutralizing a potential CaMKII phosphorylation of a serine at position 525 (S525A; corresponding to S471 in mouse ANO1-*ac*; Figure 4c), located in the first intracellular loop, led to large currents that were similar to that produced by the S727A mutant. Second, the expression of a phosphomimetic mutant at S525D led to smaller currents than those produced by the S525A mutation. These data would suggest that CaMKII may also phosphorylate S525.

Ayon *et al.* [177] explored the regulation of mouse ANO1-*a* expressed in HEK-293 cells by endogenous CaMKII and the Ca^2+^-independent serine-threonine phosphatases PP1/PP2A. Currents produced by the expression of ANO1 ran down ~ 65% from their initial level in cells dialyzed with 500 nM Ca^2+^ and 5 mM ATP (Figure 4a). In contrast, removing ATP obliterated the initial current rundown. After 20 min of cell dialysis, ANO1 currents were ~ 3-fold larger in cells dialyzed with no ATP vs. cells loaded with ATP (data not shown). These results were consistent with an ATP-dependent down-regulation of channel activity and argued against the proposed permissive role of ATP to support channel activity [161]. The rundown of ANO1 was partially attenuated by inhibiting CaMKII with KN-93 or the peptide inhibitor ARIP. Blocking PP1/PP2A with okadaic acid or cantharidin led to rundown of ANO1 in the absence of ATP. These data suggested that CaMKII-mediated phosphorylation inactivated ANO1, and a role for PP1/PP2A could only be revealed when phosphorylation was limited by omitting internal ATP. These observations were remarkably similar to those made for native CaCCs in smooth muscle cells (Figure 4b) [54–56,59,84]. Additionally, Ayon *et al.* [177] carried out site-directed mutagenesis to identify one or several potential sites for CaMKII phosphorylation. Of four sites bearing the consensus CaMKII sequence RxxS/T, only the S528A (identical to S525 in Lin *et al.* [176] and corresponding to S471 in mouse ANO1-*ac*; see Figure 4c; the serine at position 528 in Ayon *et al.* [177] was relative to the full mouse ANO1 sequence including exon 0: NCBI sequence: XP_036008438.1) mutant displayed attenuated rundown that was similar to that produced by either one of the two CaMKII inhibitors on wild-type ANO1, suggesting that S528 is one possible site responsible for CaMKII-induced inactivation of ANO1 (Figure 4c).

Finally, a more recent report by Ko *et al.* [178] investigated the crosstalk between the regulation of the *ac* variant of mouse ANO1 expressed in HEK-293 cells by CaMKII phosphorylation and the membrane phospholipid PIP_2_ (discussed in the next section). Similar to Ayon *et al.* [177] they found that omitting ATP, or replacing ATP with AMP-PNP, led to currents that were ~ 2.5–4.5 times larger than those measured in cells dialyzed with 3 mM ATP. While wild-type ANO1 was insensitive to inhibition of PKC, Erk, or PKA, the CaMKII inhibitor KN-62 enhanced the current. The same group also proposed S673 (Figure 4c; same site identified by Lin *et al.* [176]) as the serine residue phosphorylated by CaMKII because of the three sites investigated, only the S673A mutant displayed augmented currents in the presence of ATP, and only the phosphomimetic mutant S673D exhibited reduced currents in the absence of ATP. Finally, noise analysis of wild-type ANO1 currents inactivated in the presence of ATP revealed that single-channel currents were significantly reduced by phosphorylation while the maximum open probability and channel number were unaffected.

These studies support the notion that direct phosphorylation by CaMKII of at least one of the potential serine residues largely results in the ATP-dependent inactivation of Ca^2+^-, voltage-, and time-dependent I_Cl(Ca)_ in native cells. The data also suggest that the down regulation may be linked to a partial closure of the permeation pathway. This regulatory modality was shown to influence the pharmacology of native CaCCs. For instance, Niflumic acid, a classical CaCC inhibitor known to exert weak open state channel block [179], was less potent at inhibiting I_Cl(Ca)_ in rabbit PASMCs under conditions promoting global phosphorylation and channel closure [58]. Since permeation and gating are tightly linked, it will be of interest to determine the impact of phosphorylation on single channel conductance when other more permeant anion species such as I^−^ or SCN^−^ serve as charge carriers. It is important to emphasize that the regulation of ANO1 by CaMKII-induced phosphorylation is not the only mechanism involved in current rundown. Patch excision in the inside-out configuration leads to the rapid rundown of ANO1 channels in the absence of CaMKII and ATP in the bathing solution [175]. This rundown may be due to a combination of factors including the loss of essential factors such as phosphatidylinositol-(4,5)-bisphosphate (see next section), loosely bound ancillary subunits, and perturbations of the microenvironment surrounding ANO1 (caveolae, actin cytoskeleton, etc.).

## Regulation of ANO1 by phosphatidylinositol-(4,5)-bisphosphate and other membrane lipids

Phosphatidylinositol-(4,5)-bisphosphate (PIP_2_), a phospholipid located in the cytoplasmic leaflet of the plasma membrane, composes ~ 1% of the total acidic lipids in the membrane [180,181]. Although this phospholipid constitutes a small fraction of the total membrane lipid composition, it is the most abundant phosphoinositide (> 99%) [180]. PIP_2_ regulates a plethora of ion channels, transporters, and numerous signal transduction pathways [181,182]. In view of the importance of this signaling molecule in modulating channel function, it is not surprising that investigators quickly began exploring the possibility that PIP_2_ may also modulate Anoctamins as summarized below.

**Figure 5.**
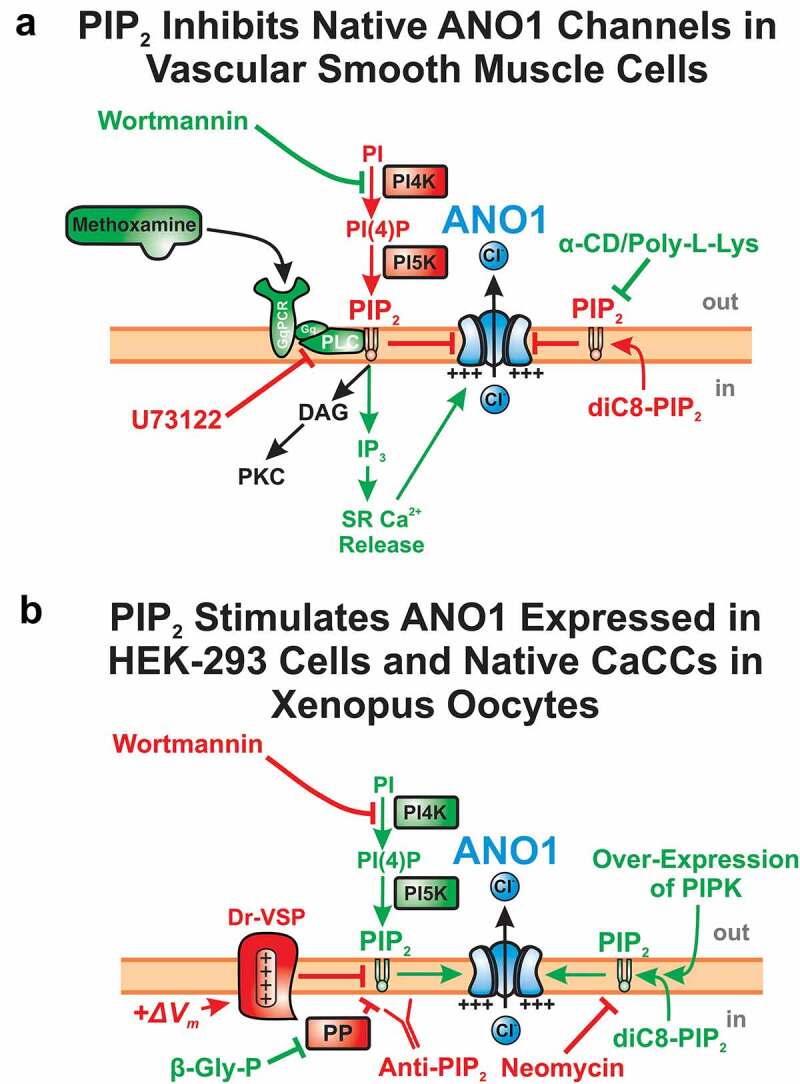
Contrasting effects of the membrane phospholipid phosphatidyl-(4,5)-bisphosphate (PIP_2_) on native ANO1-encoded CaCCs and ANO1 expressed in HEK-293 cells. (a and b) These two illustrations show the experimental strategies used to determine the effects of PIP_2_ on membrane currents associated by ANO1. Pharmacological agents, pathways and signaling molecules ultimately leading to inhibition or stimulation of ANO1 are respectively labeled in red or green. (a) At higher concentrations (tens of μM), Wortmannin inhibits phosphatidylinositol-4-kinase (PI4K) and blocks the biosynthesis of PIP_2_, leading to stimulation of ANO1. Stimulation of G_q_-Protein Coupled Receptor (GqPCR) by the α_1_-adrenergic receptor agonist methoxamine leads to activation of phospholipase C (PLC), which breaks down PIP_2_ into diacylglycerol (DAG), an endogenous activator of protein kinase C (PKC), and inositol trisphosphate (IP_3_), which stimulates ANO1 by elevating intracellular Ca^2+^ levels by triggering Ca^2+^ release from the sarcoplasmic reticulum (SR). In vascular smooth muscle cells, activation of the latter pathway would open ANO1 through both direct stimulation by Ca^2+^ and relief of PIP_2_ inhibition on ANO1. The panel also shows that ANO1 could be stimulated by α-cyclodextrin (α-CD) or Poly-L-Lysine (Poly-L-Lys), which respectively tightly binds or scavenges PIP_2_. On the other hand, blocking PLC with U73122 or an internal application of a soluble form of PIP_2_, diC8-PIP_2_, led to inhibition of CaCCs [167][change reference to 183]. Gq: trimeric GTP-binding protein G_q_; PI: phosphatidylinositol; PI(4): phosphatidylinositol-(4)-monophosphate; PI5K: phosphatidylinositol-5-kinase. (b) Inhibition of PIP_2_ biosynthesis with Wortmannin, enhanced degradation mediated by co-expression of *Danio rerio* voltage-sensitive (+*ΔV_m_*) phosphatase (DR-VSP), neutralization through tight binding of PIP_2_ by an internal application of a PIP_2_-specific antibody (Anti-PIP_2_) or by the positively charged Neomycin led to inhibition and accelerated rundown of ANO1. In contrast, an internal application of diC8-PIP_2_, co-expression of ANO1, Dr-VSP and phosphatidyl-inositol-kinase (PIPK), or the inhibition of protein phosphatases by the broad-spectrum blocker β-glycerophosphate pentahydrate (β-Gly-P), led to enhancement of ANO1 [142,170–173][change references to 151,186,187,188,189]

Through a combination of excised and whole-cell patch clamp electrophysiology, and biochemical and pharmacological approaches, Pritchard *et al.* [183] investigated if CaCCs in rat PASMCs, previously confirmed to be encoded by ANO1 [184,185], are regulated by PIP_2_ (Figure 5a). Intracellular application of diC8-PIP_2_, a water-soluble PIP_2_ analog, dose-dependently blocked I_Cl(Ca)_ between 0.1 and 10 μM. Consistent with this observation, I_Cl(Ca)_ increased following PIP_2_ breakdown by α_1_-adrenoceptor and PLC activation with methoxamine, PIP_2_ binding by α-cyclodextrin (a cell permeable phospholipid acceptor), PIP_2_ scavenging by poly-L-lysine, or inhibition of PIP_2_ synthesis with Wortmannin (a PI4K inhibitor at high concentrations). In contrast, PLC inhibition by U73122 to increase PIP_2_ synthesis reduced I_Cl(Ca)_. Together, these results indicated that PIP_2_ inhibits native CaCCs in vascular smooth muscle cells. Agonist-induced engagement of GPCRs coupled to PLC would be expected to exert a dual stimulatory effect by releasing Ca^2+^ from intracellular stores and relief of PIP_2_ inhibition due to its breakdown by PLC. But these results have not been replicated with expressed ANO1 as discussed below.

As opposed to the results described above in native vascular smooth muscle cells, Ta *et al.* [186] showed that ANO1 expressed in HEK-293 cells are potently stimulated (~ 5-fold) by an intracellular application of diC8-PIP_2_, an effect that was accentuated at low [Ca^2+^]_i_ (Figure 5b). In contrast, diC8-PIP_2_ modestly inhibited ANO2 (or TMEM16B), and this effect was Ca^2+^-independent. Co-expression of ANO1 and *Danio rerio* voltage-sensitive phosphatase (Dr-VSP), which depletes endogenous PIP_2_ when the membrane is depolarized [187], produced time-dependent inhibition and stimulation of ANO1 and ANO2, respectively, during steps to +100 mV. Co-expression of a PIP kinase antagonized these effects, which were undetectable when ANO1 and ANO2 were co-expressed with an inactive mutant of Dr-VSP.

A subsequent study by De Jesús Pérez *et al.* [188] showed that co-expression of ANO1 with Dr-VSP also caused time-dependent decline of ANO1 during strong depolarizing steps, again suggesting that PIP_2_ depletion inactivates the channels. Repolarization to negative potentials, which suspends Dr-VSP phosphatase activity, led to a partial recovery of ANO1 in the presence of intracellular Mg^2+^ and ATP, an observation consistent with resynthesis of PIP_2_ promoting channel activity. Moreover, an intracellular application of diC8-PIP_2_ in inside-out membrane patches from ANO1-expressing HEK-293 cells attenuated the rundown of single ANO1 channels exposed to high intracellular [Ca^2+^]_i_ (100 μM).

These investigators also examined whether fatty acids (FA), phosphatidylserine (PS), and cholesterol (discussed in the next section) regulate ANO1. They found that stearic, arachidonic, oleic, docosahexaenoic, and eicosapentaenoic fatty acids, and methyl stearate and PS all inhibit ANO1 in a concentration- and voltage-dependent manner. These effects were attributed to a direct membrane-delimited lipid-channel protein interaction and were not associated with PIP_2_ depletion or a change in the membrane distribution of ANO1 due to altered trafficking.

In another study, Tembo *et al.* [151] investigated the regulation by PIP_2_ of endogenous CaCCs in *Xenopus* oocytes (Figure 5b), which are known to be the product of ANO1 [100]. Similar to the two studies described earlier on expressed ANO1, these investigators concluded that ANO1 gating requires both Ca^2+^ and PIP_2_. ANO1 channels quickly ran down in the presence of 2 mM Ca^2+^ applied on the internal side of the membrane, and this process was partially reversed by exposure to diC8-PIP_2_ on the cytoplasmic side of the membrane. Additionally, an internal application with a specific PIP_2_ antibody or the PIP_2_ scavenger neomycin accelerated current decay, whereas supplying ATP or blocking phosphatase activity with the wide-spectrum inhibitor β-glycerophosphate pentahydrate delayed current rundown (Figure 5b).

Centeio *et al.* [189] documented that an internal application of diC8-PIP_2_ robustly stimulated over-expressed ANO1 currents in HEK-293 cells but produced little to no effect on endogenous ANO1 currents in HT_29_ colonic epithelial cells. They proposed that expressed ANO1 channels may have better accessibility to intracellular Ca^2+^ than those in a native environment. Studies on cloned ANO1 channels unequivocally demonstrated a stimulatory effect of PIP_2_ on expressed ANO1, whereas the regulation of native ANO1 channels in some mammalian cells remains unclear and appears to be cell- and condition-dependent.

Studies on the molecular basis for the PIP_2_ effects have used mutagenesis and molecular dynamics simulations to identify several regions that could bind PIP_2_ and modify channel behavior. Le *et al.* [150] proposed grouping the pore and gating region into a PIP_2_ binding regulatory module (TMD3-TMD5) that is linked to channel inactivation and a Ca^2+^-binding gating module (TMD6-TMD8) that is involved in channel activation (Figure 3g). They identified 6 basic residues near the cytosolic interface of TMDs 3–5 that form a putative PIP_2_ binding site, R451, K4561, R482, K567, R575, and K579 (mouse ANO1-*a* variant). Molecular dynamics simulations suggested that PIP_2_ binding to this site in the Ca^2+^ bound channel causes TMD4 to move away from TMD6 to contribute to a fully open pore [153]. Yu *et al.* [190] identified three potential PIP_2_ binding sites with the first located on the cytosolic side of TMD1-2 near the inter-dimer space, the second at the base of TMD6, and the third on the intracellular loop between TMD2-3 (Figure 3f). This group suggested that PIP_2_ binding to these sites may influence channel activity through modulation of Ca^2+^ binding, channel gating, or modification of the ion conduction pathway. As mentioned previously, Ko *et al.* [178] proposed that PIP_2_ differentially regulates ANO1 segments “*a*” and “*ac*”. They suggested that CaMKII phosphorylates S673 (Figure 4c) on the third intracellular loop, which causes an allosteric modulation of the first intracellular loop, imparting differential sensitivities to PIP_2_ for the splice variants in the presence of intracellular ATP. These studies demonstrated that there is a multiplicity of functional PIP_2_ binding sites, but a comprehensive understanding of the modulation of the channel by PIP_2_ binding and how this interaction is influenced by of CaMKII-mediated phosphorylation is still lacking.

## Is ANO1 located in membrane lipid rafts and caveolae?

Compartmentalization of signaling pathways is an important mechanism for driving cell responses to accomplish specific tasks dictated by unique stimuli. Confinement of ion channels, receptors, and enzymes to lipid rafts and caveolae is a good example of subcellular functional fine tuning [191,192]. Sones *et al*. [193] first investigated whether the biophysical properties of CaCCs in murine portal vein smooth muscle were affected by methyl-β-cyclodextrin (MβCD), a cholesterol depleting agent commonly used as a tool to disrupt membrane cholesterol- and sphingolipid-enriched structures called lipid rafts, and caveolae, a subset of lipid rafts [191]. The study showed that a 5-min exposure to MβCD (3 mg/mL) enhanced the maximal conductance of CaCCs and slightly increased their voltage sensitivity. These effects were inhibited by co-administration of cholesterol in the presence of MβCD. Biochemical fractionation studies indicated that ANO1 was located in low-density fractions enriched in lipid raft markers such as flotillin-2 and caveolin and shifted to less buoyant fractions after exposure to MβCD. Although the mechanism responsible for enhancing CaCC activity following cholesterol depletion remains to be clarified, these experiments suggested that at least a fraction of ANO1 channels traffics to caveolae in vascular myocytes, where they could hypothetically participate in compartmentalized membrane signaling. This question was revisited by De Jesús-Pérez *et al*. [188] on ANO1 expressed in HEK-293 cells. They too found that acute exposure to MβCD enhanced ANO1, but the effect was transient. Longer incubations with MβCD (30 min) delayed the ANO1 rundown following membrane rupture but did not alter the steady-state level reached after 5 min. An exogenous addition of cholesterol antagonized the effects of MβCD. The authors speculated that the activity of MβCD on ANO1 did not appear to be related to changes in PIP_2_ levels. They proposed that similar to free fatty acids and phosphatidylserine, cholesterol may regulate ANO1 through direct lipid–protein interactions. Consistent with this idea was the report of Malvezzi *et al.* [140] showing that the dual scramblase/ion channel activity of the purified ancestral Anoctamin afTMEM16 reconstituted in lipid liposomes was very sensitive to the lipid composition. When reconstituted in liposomes containing a mixture of 1-palmitoyl-2-oleoyl phosphatidylglycerol and 1-palmitoyl-2-oleoyl phosphatidylglycerol afTMEM16 activity was inhibited, whereas a mixture of *E. coli* polar lipids and egg phosphatidyl choline supported afTMEM16 function.

## Is ANO1 Regulated by the cytoskeleton?

Participation of the actin cytoskeleton and microtubules in the gating of native Cl^−^ channels recorded in bronchial epithelial cells had been suggested prior to the discovery of Anoctamins [194]. A recent study on expressed ANO1 showed that actin depolymerization with cytochalasin D or actin stabilization with phalloidin suppressed the current [161]. This was a surprising finding considering that stabilizing and destabilizing the actin cytoskeleton produced the same effect. It is possible that these pharmacological agents have direct effects on ANO1. A more recent report showed that cytochalasin D had no effect on the amplitude of ANO1-encoded endogenous CaCCs in mouse portal vein smooth muscle cells, but slowed the deactivation kinetics. However, the application of jasplakinolide, an agent promoting actin polymerization, inhibited these effects [195]. The same study revealed that cytochalasin D produced no effect on currents resulting from the expression of various combinations of splice variants of mouse ANO1 in HEK-293 cells. They reasoned that the lack of effect of this agent could be attributed to the better developed actin cytoskeleton in muscle vs. non-muscle cells, and that such experiments argued in favor of a true interaction of the actin cytoskeleton in myocytes as opposed to a nonspecific and direct effect of cytochalasin D on ANO1. Support for the hypothesis of a physiological interaction of the cytoskeleton with ANO1 also came from results of a proteomic strategy, which showed that ANO1 physically interacts with a network of scaffolding proteins (ezrin, radixin, moesin, and RhoA) that links the plasma membrane to the actin cytoskeleton [196]. ANO1 co-localized with moesin, ezrin, radixin when expressed in HEK-293 cells, as well as in the apical membrane and intercalated excretory ducts of salivary glands. Knockdown of moesin by shRNA reduced ANO1 currents. This effect was not due to a reduction of the amount of ANO1 at the plasma membrane. As a whole, these studies provided a fragmentary picture on how the cytoskeleton may regulate ANO1 and further analysis is warranted.

## Regulation through coupling of ANO1 with other membrane proteins

Many pore-forming α subunits of ion channels have ancillary or accessory subunits (usually labeled β, γ, δ, etc.) that physically interact with the α subunit to regulate their translocation to the membrane, compartmentalization to a membrane subdomain that conveys specific localized functions, and biophysical and pharmacological properties. A report by Perez-Cornejo *et al.* [196] identified a large network of proteins directly or peripherally associated with ANO1 that have the potential to fine tune its properties and function. As an example, one of the identified proteins was PI4Kα, a kinase catalyzing a key step in the biosynthesis of PIP_2_. Although it is not a true β subunit, its presence in the microdomain of ANO1 certainly could play an important role in modulating its gating by maintaining PIP_2_ levels in the local lipid bilayer environment supporting the channel. The subsections below describe recent literature highlighting unique interactions of specific proteins with ANO1 by altering its expression, behavior, or physiological role in different cell types.

### CLCA1 and 2

As discussed in a previous section, the CLCA family of proteins was the first class of proteins proposed as molecular candidates for native CaCCs [89,102,103,197], but the idea that they are pore-forming subunits supporting CaCC activity was later dismissed [102,107]. The possible role of CLCA proteins, particularly hCLCA1 and 2, as regulators of CaCC expression and function, was revisited after the discovery of Anoctamins. hCLCA1 and its mouse ortholog mCLCA3, like many other members of this protein family, are soluble proteins secreted by airway and gut epithelial cells [105]. Expression of hCLCA1 in two mammalian cell lines led to stimulation of endogenous CaCCs [198–201]. Yurtsever *et al.* [199] first showed that this process involved the proteolytic self-cleavage of hCLCA1 due to the presence of a novel zincin metalloprotease domain at its N-terminal end. The stimulation of CaCCs was not linked to the proteolytic activity of hCLCA1 *per se* because exposure to an N-terminal mutant fragment lacking enzymatic activity still led to enhanced CaCC activity. An ensuing study from Sala-Rabanal *et al*. [200] demonstrated that hCLCA1 secreted from cells increased endogenous ANO1-induced I_Cl(Ca)_ in HEK-293T cells through a paracrine mechanism. This effect, which occurred within minutes, was not due to increased expression of ANO1 but rather an increase in the number of channels on the cell surface. This occurred by stabilization of ANO1 at the plasma membrane, most likely by decreasing internalization. Subsequent reports demonstrated that the von Willebrand factor type A (vWF) domain located in the N-terminus of hCLCA1 is responsible for the interaction with ANO1 [201,202]. The engagement of ANO1 was speculated to involve Mg^2+^-dependent binding of the extracellular loop between TMD9 and 10 of ANO1 to a conserved metal ion-dependent adhesion site (MIDAS) motif on the vWF domain of hCLCA1. Finally, the effects described above are not unique to hCLCA1 as the expression of hCLCA2 in a HEK-293T cell line stably expressing hANO1 nearly doubled the magnitude of I_Cl(Ca)_ evoked by the Ca^2+^ ionophore ionomycin [203].

### Local functional coupling of ANO1 with other ion channels

Recent evidence has shown that in small nociceptive neurons of dorsal root ganglia, ANO1 channels may be confined to lipid raft domains, where they structurally colocalized and functionally interacted with GPCRs and IP_3_ receptors (IP_3_R) [204]. In the majority of these neurons, Ca^2+^ entry through VGCCs was unable to activate ANO1. In contrast, stimulation of type 2 bradykinin or type 2 protease-activated receptors triggered a robust stimulation of ANO1 currents produced by Ca^2+^ mobilization through IP_3_R located in regions of the endoplasmic reticulum that made close contacts with the plasma membrane. The tight physical coupling of ANO1 with IP_3_R involves tethering of the C-terminal end and the first intracellular loop of ANO1 with one or more undefined regions of IP_3_R. Cholesterol depletion and the consequent destruction of lipid rafts uncoupled GPCRs, ANO1, and IP_3_R. Cholesterol depletion also unmasked the stimulation of ANO1 by Ca^2+^ influx through VGCCs. This is consistent with the evidence discussed earlier showing that ANO1 may be located in caveolae [193]. There is speculation that the close association of ANO1 with IP_3_R allows for shaping specific localized Ca^2+^ signals in response to inflammatory signals rather than requiring global changes in [Ca^2+^]_i_. This arrangement would help minimize the potentially negative impact of self-sustaining positive feedback loops between ANO1 and VGCCs that could lead to neuronal hypersensitivity [205].

A similar relationship was documented in airway smooth muscle cells where CaCCs were shown to interact with ryanodine receptors (RyR) [86]. In these cells, spontaneous and spatially localized Ca^2+^ release events called Ca^2+^ sparks can activate clusters of BK channels and CaCCs, producing the so-called Spontaneous Transient Outward Currents (STOCs) and Spontaneous Transient Inward Currents (STICs), respectively. In some cells, both STOCs and STICs can overlap in time, creating “STOICs” [206]. Bao *et al.* [86] showed that STICs lagged Ca^2+^ sparks by only 3 ms and speculated that CaCCs were most likely all located in areas of the membrane juxtaposed with the sarcoplasmic reticulum making close contact with the former, where Ca^2+^ release through RyRs results in CaCC activation. They hypothesized that such a structural arrangement is necessary to gate ANO1 because its Ca^2+^ sensitivity at physiological membrane potentials would be too low if the channels were activated by global increases in [Ca^2+^]_i_. Subsequent studies showed that ANO1 is responsible for I_Cl(Ca)_ in airway myocytes and is involved in smooth muscle contraction to agonists and airway hypercontractility in asthma [207–212]. More studies using biochemical (co-immunoprecipitation and Western blot, peptide displacement) and advanced microscopy (super resolution nanomicroscopy, proximity ligation assays, Förster Resonance Energy Transfer) techniques are needed to determine if the interaction of ANO1 and RyR requires direct physical tethering between the two proteins or involves intermediary scaffolding proteins.

ANO1 also physically couples to Transient Receptor Potential (TRP) channels, particularly with at least two members of the vallinoid subfamily, TRPV1 and TRPV4, and two members of the canonical TRP subfamily, TRPC1 and TRPC6. Takayama *et al.* [213] showed that in the apical membrane of epithelial cells of choroid plexus (CPECs), ANO1 structurally and functionally interacts with heat-sensitive Ca^2+^-permeable TRPV4, a property not shared by ANO4, ANO6, or ANO10, which are also expressed in CPECs. This interaction could also be recapitulated by co-expression of the channels in HEK-293 cells. ANO1-mediated I_Cl(Ca)_ could be activated in wild-type CPECs by combining warmth and hypo-osmotic medium, which are endogenous activators of TRPV4, or the specific TRPV4 agonist, GSK1016790A. ANO1 currents were not activated in TRPV4-KO CPECs.

In this system, local Ca^2+^ entry through TRPV4 stimulates neighboring ANO1 leading to Cl^−^ efflux due to an outwardly directed electrochemical Cl^−^ gradient. Cl^−^ efflux through ANO1 and the K^+^- Cl^−^ cotransporter (KCC), and concomitant K^+^ efflux through this exchanger, drives the osmotic exit of water through aquaporin 1/4 channels and a reduction in cell volume. This tight TRPV4-ANO1 coupling is thought to play a key role in maintaining fluid balance in the brain by promoting ventricular drainage and cerebral fluid movement.

A similar type of coupling between ANO1 and TRPV4 was reported in exocrine acinar cells of the salivary and lacrimal glands [214]. In these systems, stimulation of Ca^2+^ influx through TRPV4 by a muscarinic agonist or heat, reinforced by IP_3_R-mediated Ca^2+^ release, stimulates Cl^−^ efflux through ANO1. This stimulation leads to cell shrinkage (so-called “regulatory volume decrease” mechanism following the initial cell swelling) by passive water efflux through aquaporin 5 channels. This water efflux is the mechanism involved in saliva or tear production [215]. This study also showed that ANO1 could be activated by store-operated Ca^2+^ entry (SOCE) through TRPC1. In TRPC1 KO cells I_Cl(Ca)_ triggered by SOCE were potently inhibited. In contrast, ANO1 did not couple to TRPC3. Whether ANO1 and TRPC1 are physically associated was not determined. These studies beg the following question: Are the ANO1 channels interacting with TRPV4 the same ones functionally coupling with TRPC1, or do they represent different subpopulations? In cerebral artery smooth muscle cells, ANO1 was shown to interact with TRPC6. This mechanism was suggested to amplify the vasoconstriction elicited by Hyp9, a selective activator of TRPC6 [216]. Co-immunoprecipitation and FRET revealed that ANO1 and TRPC6 reside within the same macromolecular complex.

Activation of TRPV1 by noxious stimuli such as capsaicin (CAP) in sensory neurons is critically involved in pain sensation. This pathway causes membrane depolarization and activation of voltage-gated Na^+^ channels and action potential generation. Takayama *et al.* [217] showed that ANO1 gating primarily mediated depolarization in response to Ca^2+^ entry through TRPV1, which is physically bound to ANO1 in these cells. In addition, the TRPV1-ANO1 coupling led to enhanced action potential firing in response to CAP, a response that was also blocked by the ANO1 blocker T16_Inh_-A01. Shah *et al.* [218] reported a similar interaction between TRPV1 and ANO1 in peripheral somatosensory neurons. They also showed that the CAP-induced ANO1 conductance involved ER Ca^2+^ release through an additional interaction with type 1 IP_3_R (IP_3_R1). Proximity ligation assays and super-resolution nanomicroscopy confirmed that TRPV1, ANO1, and IP_3_R1 reside in the same microdomain. These results are consistent with the idea that the functional interaction between the three ion channels primarily occurs within tight submembrane compartments formed between ER domains in close proximity with the plasma membrane (~ 20–30 nm). Such coupling supports localized Ca^2+^ signaling, which amplifies nociception.

### KCNE1 – A novel ancillary β subunit regulating ANO1?

A provocative report published in 2021 by Ávalos Prado *et al.* [219] revealed a novel interaction between KCNE1 (also referred to as MinK), one of five K^+^ channel auxiliary subunits (KCNE1-5), and ANO1. KCNE1 associates with the delayed rectifier K^+^ channel KCNQ1 to produce the slow delayed rectifier K^+^ current in cardiac myocytes called I_Ks_. Mutations in KCNE1 and KCNQ cause cardiac arrhythmias due to Long QT syndrome [220]. Co-expression of KCNE1 and ANO1 in HEK-293 T cells converted ANO1-induced I_Cl(Ca)_ to voltage-dependent Cl^−^ currents lacking Ca^2+^-sensitivity. This interaction was noted in proximal convoluted tubular cells indicating that it also prevails in native cells. The investigators used a single molecule pulldown assay and photobleaching step strategy to confirm that KCNE1 is directly bound to ANO1 at its N-terminus with a stoichiometry of 2 KCNE1 subunits associated with 2 ANO1 monomers. Furthermore, expression of KCNE1 with the double ANO1 mutant I637A and Q645A (located in TMD6), clinically relevant inherited mutations, led to activation of ANO1 in the absence of internal Ca^2+^, which abolished the modulation of ANO1 by KCNE1 [169]. This finding led to the speculation that KCNE1 mimics the effects of the double mutant in TMD6, conferring voltage-dependent gating in the absence of Ca^2+^. Finally, of the four other KCNE subunits tested, only KCNE5 exerted similar effects on ANO1. These results indicate that KCNE1 (and perhaps KCNE5) are regulatory β subunits of ANO1 because: 1) when expressed alone, KCNE1 does not produce an ion conductance; 2) KCNE1 physically interacts with ANO1 in a fixed stoichiometry, which profoundly modulates the biophysical properties of ANO1; and 3) the physical and functional interaction of KCNE1 and ANO1 was also found in native cells. This publication paved a new and exciting era for discovery, with significant translational potential since mutations of KCNE proteins have been associated with many pathologies.

## Summary and concluding remarks

The goal of this review was to provide a snapshot of our current understanding how CaCCs encoded by ANO1 are regulated by various signaling modalities (summarized in Table 1). The discovery of a valid molecular candidate for small conductance voltage- and time-dependent CaCCs took a painstakingly slow, meandrous, and often confusing path. We hope that tackling this topic from an historical perspective that included earlier work on native CaCCs will enable the reader to eventually connect the dots as to how native CaCCs and ANO1 are correlated and regulated *in vivo*. After the discovery of TMEM16 genes, the development of new molecular tools, techniques, experimental strategies, and animal models convincingly showed that the original 8 TMD protein model (Figure 2a) that led to the “Anoctamin” acronym necessitated a revision to a 10 TMD topology (Figure 2c), which is now the model universally accepted thanks to seminal structural studies on several members of this family. We now know that ANO1 channels assemble as homomeric dimers, in which each monomer forms a narrow hourglass-shaped anion permeation pathway, and each monomer functions independently from each other. Evidence suggests that ANO1 activation does not require binding of mobile or tethered Ca^2+^-CaM, ATP binding, or a phosphorylation step mediated by CaMKII. Instead, multiple studies support the concept of activation by direct binding of at least two calcium ions per monomer in a region between TMD6 and TMD8 located within the transmembrane electric field. The mechanism of ANO1 gating is further complicated by compelling evidence showing that an acidic region within the 1^st^ intracellular loop between TMD1 and 2, and alternative splicing within the N-terminal end (splice variants *a* and *b*) and 1^st^ intracellular loop (splice variants *c* and *d*; Figure 2c), modulate the pharmacology, and Ca^2+^-, time- and voltage-dependence of the channel in profound ways. The cryo-EM models that are presently available show that portions of the N-and C-terminus are unstructured. In the cell, these domains are likely to interact with intracellular loops and play a role in channel gating.

**Table 1.** Summary of the major effects of several modulators and experimental strategies employed to determine their role in regulating native and expressed ANO1

Modulator	Effects on ANO1 Current	Native ANO1	Exp. ANO1	Cell Type	Experimental Approaches	Refs
ATP	Promotes channel closure or inactivation	√		Rabbit and rat PASMCs, equine tracheal SMCs	Cell dialysis; rundown	55, 57, 58, 59, 84, 221
			√	HEK-293 cells	Cell dialysis; rundown	75, 177, 178
	Promotes channel opening		√	HEK-293 cells	Internal application of ATP to inside-out patches; apyrase (ATP cleaving enzyme)	161
0 ATP or AMP-PNP	Promotes channel opening	√		Rabbit PASMCs	Cell dialysis; attenuated rundown and runup	57, 58, 59, 84
			√	HEK-293 cells	Cell dialysis; attenuated rundown and runup	177, 178
CaM	No role		√	HEK-293 cells	W7 (CaM inhibitor); internal application of wild-type and mutant CaM; site-directed mutagenesis of speculated CaM binding site; vesicular Cl^−^ transport assays with purified ANO1 and CaM; over-expression of Ca^2+^-insensitive CaM mutants; Ba^2+^, which activates ANO1, does not influence CaM	155, 165, 166
	Enhances Ca^2+^ sensitivity at 1 μM [Ca^2+^]_i_; decreases channel activity at [Ca^2+^]_i_ > 10 μM		√	HEK-293 cells	Demonstration of pre-association of CaM using ChIMP assay; site-directed mutagenesis of CaM; Ca^2+^-dependence of ANO1	167, 168
	Is required for ANO1 channel opening in the presence of internal ATP		√	HEK-293 cells	TFP and J-8 (CaM inhibitors); internal application of CaM to inside-out patches	161
	Promotes channel opening at submicromolar [Ca^2+^]_i_; rundown of ANO1 current at supraphysiologic [Ca^2+^]_i_		√	HEK-293 cells	Truncation of the CaM binding domain (RCMB); internal application of peptides corresponding to RCMB domain	162
CaMKII	Promotes channel closure or inactivation	√		Equine tracheal SMCs	W7; KN-93 and ARIP (CaMKII inhibitors); intracellular AMP-PNP	84
		√		Rabbit coronary and pulmonary SMCs	KN-93 and ARIP; AutoCaMKII	54, 55
		√		Cultured mouse basilar artery SMCs	CaMKII siRNA; S727A mutation and S727D phosphomimetic mutation of ANO1	176
			√	HEK-293 cells	KN-62; KN-93; ARIP; S528A and S673A mutations of mANO1; S673D phosphomimetic mutation of ANO1	75, 177, 178
CaN/PP2B	Promotes channel opening	√		Rabbit coronary and pulmonary SMCs	Cyclosporin A (CsA); CaN peptide inhibitor; exogenous CaNA-α	55, 56, 57
PP1/PP2A	Promotes channel opening in the absence of intracellular ATP	√		Rabbit PASMCs and equine tracheal SMCs	Okadaic acid (PP1/PP2 blocker); cantharidin (PP1/PP2 blocker); CsA	57, 58, 84
			√	HEK-293 cells	Okadaic acid; cantharidin	177
PP1	Promotes channel opening in the absence of intracellular ATP	√		Rabbit PASMCs	NIPP-1 (PP1 peptide inhibitor); fostriecin (specific PP2A inhibitor): no effect	57
PIP_2_	Decreases ANO1 current	√		Rat PASMCs	Intracellular application of DiC8-PIP_2_; methoxamine (PLC activation); α-cyclodextrin (binds PIP_2_); poly-L-lysine (PIP_2_ scavenger); wortmannin (PI4K inhibitor); U73122 (PLC blocker)	183
	Increases ANO1 current		√	HEK-293 cells	Intracellular application of DiC8-PIP_2_; co-expression of Dr-VSP; wortmannin	186, 188, 189
		√		Xenopus oocytes	Anti-PIP_2_ antibody; neomycin (PIP_2_ scavenger); β-glycerophosphate pentahydrate (protein phosphatase inhibitor); expression of PIPK	151, 189
	No effect	√		HT29 colonic epithelial cells	Intracellular application of DiC8-PIP_2_	189
Cholesterol	Decreases ANO1 current	√		Mouse portal vein SMCs	MβCD (membrane cholesterol depleting agent); exogenous cholesterol	193
			√	HEK-293 cells	MβCD (membrane cholesterol depleting agent); exogenous cholesterol	188
Free Fatty Acids and PS	Decrease ANO1 current		√	HEK-293 cells	Exogenous applications	188
Actin Cytoskeleton	Increases or supports ANO1 current		√	HEK-293 cells	Cytochalasin D (actin depolymerization); phalloidin (actin stabilizer)	161
	No effect on amplitude; slower deactivation kinetics	√		Mouse portal vein SMCs	Cytochalasin D; phalloidin; jasplakinolide (promotes actin polymerization) opposed effects on kinetics	195
	No effect		√	HEK-293 cells	Cytochalasin D	195
Moesin	Increases ANO1 current		√	HEK-293 cells	shRNA	196
CLCA1/2	Increases ANO1 through stabilization at the plasma membrane	√	√	HEK-293 cells	Co-expression and colocalization studies; exogenous application of CLCA wild-type and truncated protein fragments; nocodazole (inhibitor of microtubule-dependent internalization); siRNA; truncation studies by site-directed mutagenesis	199, 200, 201, 203
KCNE1/5	Confers voltage-dependent gating in the absence of Ca^2+^		√	HEK-293 cells	Co-expression and colocalization studies; single molecule pulldown assays; truncation studies by site-directed mutagenesis; KCNE1 knockdown; siRNA	219
		√		Proximal convoluted tubular cells	KCNE1 knockdown; siRNA; Angiotensin II exposure (simulates KCNE1-mediated effects on ANO1)	

Nomenclature: ANO1 (Anoctamin-1); Exp. ANO1: expressed ANO1; Refs: references; ATP: adenosine triphosphate; 0 ATP: no internal ATP; AMP-PNP (adenosine 5′-(β,γ-imido)triphosphate): non-hydrolyzable analogue of ATP; SMCs; smooth muscle cells; PASMCs: pulmonary artery smooth muscle cells; CaM: calmodulin; [Ca^2+^]_i_: intracellular Ca^2+^ concentration; ChIMP assay: channel inactivation induced by membrane-tethering of an associated protein; TFP: trifluoperazine; RCMB: Regulatory Calmodulin-Binding Motif; ARIP: autocamtide-2-related inhibitory peptide; CaMKII: calmodulin-dependent protein kinase II; AutoCaMKII; constitutively active form of CaMKII; siRNA: silencing ribonucleic acid; CaN/PP2B: calcineurin/protein phosphatase 2B; CaN-A: calcineurin type A-α; PP1/PP2A; protein phosphatase 1/protein phosphatase 2A; PIP_2_: phosphatidylinositol-(4,5)-bisphosphate diC8-PIP_2_: phosphatidylinositol-(4,5)-bisphosphate diC8 (PI(4,5)P_2_ diC8); PLC: phospholipase C; PI4K: phosphatidylinositol 4-kinase; InsP4: inositol-(1,3,4,5)-tetrakisphosphate; Dr-VSP: *Danio rerio* voltage-sensitive phosphatase; MβCD: methyl-β-cyclodextrin; PS: phosphatidyl serine; shRNA: short hairpin ribonucleic acid.

In whole-cell and excised patch clamp experiments, ANO1 expressed in mammalian cell lines runs down in the presence or absence of internal ATP. Multiple mechanisms may be involved in this process including a possible intrinsic pore restriction [141,175] or Ca^2+^ desensitization by CaM-dependent inactivation at supraphysiological Ca^2+^ concentrations [167,168], CaMKII-mediated phosphorylation at low to intermediate [Ca^2+^]_i_ (≤ 1 μM), loss or biodegradation of PIP_2_ or other membrane lipids, and likely others (e.g., disruption of the cytoskeleton during patch excision). We still have a poor understanding of their relative contribution under various conditions and how they intertwine. Evidence suggests that a portion of this rundown is mediated by at least one phosphorylation step mediated by CaMKII, either in the 1^st^ intracellular loop or near the putative Ca^2+^ binding sites. This process is opposed by the serine/threonine phosphatases Calcineurin and PP1/PP2A under certain conditions (Figure 4c). Observations on cloned ANO1 remarkably replicated findings previously made for CaCCs recorded in vascular smooth muscle cells (Figure 4a and b). As for many other classes of ion channels, the membrane phospholipid PIP_2_ also regulates ANO1 (Figure 5), but a few limited studies suggest that regulation of ANO1 by PIP_2_ in native mammalian cells may be different than in overexpression systems. This could reflect differences in binding partners or membrane lipid composition. ANO1 activity is constrained by long chain saturated and unsaturated fatty acids, whereas cholesterol depletion, which destroys lipid rafts and caveolae, enhances its gating. ANO1 is directly associated or is in close proximity with proteins linked to the actin cytoskeleton. There is mounting evidence that in most if not all cell types where it is endogenously expressed, ANO1 is located in restricted spaces optimized for compartmentalized signaling, where it structurally and functionally interacts with other ion channels in the plasma membrane (TRPCs, TRPVs) and ER (IP_3_R, RyR) to produce very specific responses (secretion, cell volume regulation, excitation, others), and perhaps within the same cell. We have also learned that ANO1 can associate with hCLCA1 and hCLCA2 to regulate its trafficking and stability in the membrane. Finally, KCNE proteins may be the first canonical class of proteins obeying the clear definition of auxiliary β subunit protein partners of ANO1, modulating its biophysical properties to achieve specific cellular functions.

Despite the amazing breakthroughs made in a relative short time span since their discovery, we have only scratched the surface regarding our understanding of the biophysical properties of Anoctamins in their native environment. So many questions remain unanswered. Being in a macromolecular complex interacting with the actin cytoskeleton as well as extracellular matrix proteins, is ANO1 directly or indirectly mechanosensitive? Is it modulated by integrin and/or dystrophin complexes? Can it be associated with other members of the CLCA family of proteins to regulate its translocation and function?

It is hypothesized that the down regulation of ANO1 by CaMKII-mediated phosphorylation serves as a functional break to the sustained depolarization maintained by the positive feedback loop between Ca_V_1.2 (or any Ca^2+^ permeable voltage-dependent ion channel coupled to ANO1; e.g., TRPV1 and TRPV4) and ANO1. What is the evidence that such a mechanism regulates function (contraction, secretion, etc.) in intact excitable tissues such as smooth muscle, and *in vivo*? I_Cl(Ca)_ in rabbit arterial smooth muscle cells exhibit massive rundown (70–80% within 5 min; Figure 4b) [57–59], whereas those in recorded in rat [221] or mouse (Leblanc, unpublished observation) pulmonary artery smooth muscle cells run down much less (~ 20% or not at all); surprisingly, mouse ANO1 expressed in HEK-293 cells (Figure 4a) exhibits a phenotype that is very similar to I_Cl(Ca)_ in rabbit myocytes (Figure 4b) [177]. The reasons for such diff erences are unclear. However, they are likely due to the combination of many factors, including the exact composition and level of expression of CaMKII, Calcineurin and PP1/PP2A, the molecular architecture of the microdomain comprising ANO1, the splice variants of ANO1 that are expressed, whether KCNE1 or 5 is co-expressed with ANO1, and many other possibilities.

Another critical unanswered question concerns the regulation of ANO1 by PIP_2_ and many of its precursors and metabolites when endogenously expressed or artificially over-expressed. Some findings are diametrically opposed, but why? The arguments presented above about possible differences in the factors and conditions affecting the regulation of ANO1 by CaMKII also apply. The expression of the enzymes involved in the biosynthesis (PI4K, PI5K, etc.) and degradation (PLC, PTEN, etc.) of PIP_2_ in native cells and mammalian cell lines used to express ANO1 are likely very different. Importantly, the amount of ANO1 expressed in cell lines is much higher than in native cells, which increases the ANO1:PIP_2_ ratio. Also, if PIP_2_ stimulates ANO1 gating, why is the conductance of both native and ANO1 channels enhanced in the presence of physiological [Ca^2+^]_i_ (≤ ~ 1 μΜ) when intracellular ATP, which is the main phosphate donor in the biosynthesis of PIP_2_, is removed (Figure 4a) [57–59,177,178]? Could these seemingly discrepant results reveal dual unidentified modes of regulation by PIP_2_, inhibiting or supporting ANO1 activity depending on certain conditions, or could the regulation of KCNE proteins by PIP_2_ be responsible for these differences? Does alternative splicing alter the regulation of ANO1 by PIP_2_, and what is the reciprocal influence of CaMKII-induced phosphorylation and regulation by PIP_2_ of ANO1 *in vivo*?

CaCCs encoded by ANO1 have been implicated in many diseases such as systemic [222–225] and pulmonary hypertension [42,221,226–230], asthma [207,209,231], defective epithelial ion transport in the lungs and gut [207,232–239], and many forms of cancer [123,240–246] to name a few. More are likely to emerge in the next decade, including the discovery of ANO1 mutations directly linked to the etiology of some diseases. In most instances, ANO1 expression and function are enhanced and shown to contribute to disease initiation and/or progression. However, what are the underlying mechanisms? Are changes in membrane potential solely responsible for their activity, or do alterations in the transmembrane gradients of Cl^−^ in different compartments of the cell and organelles also playing a key role? Finally, is the regulation of ANO1 by the various signal transduction pathways discussed in this article altered and contributing to disease states? The next decade will undoubtedly be exciting as it will raise the fog on many of these questions and bring new challenging puzzles to resolve regarding the biological roles of ANO1 and its paralogs in health and disease.
